# *RTP4* is a novel prognosis-related hub gene in cutaneous melanoma

**DOI:** 10.1186/s41065-021-00183-z

**Published:** 2021-06-21

**Authors:** Yiqi Li, Jue Qi, Jiankang Yang

**Affiliations:** 1 School of Basic Medical Sciences, Dali University, Dali, 671000 Yunnan China; 2 Institute of Translational Medicine for Metabolic Diseases, Dali University, Dali, 671000 Yunnan China; 3 Department of Dermatology, First Affiliated Hospital of Kunming Medical University, Kunming, 650000 Yunnan China

**Keywords:** Melanoma, RTP4, Prognostic gene

## Abstract

**Objective:**

Melanoma accounts for 80% of skin cancer deaths. The pathogenesis of melanoma is regulated by gene networks. Thus, we aimed here to identify gene networks and hub genes associated with melanoma and to further identify their underlying mechanisms.

**Methods:**

GTEx (normal skin) and TCGA (melanoma tumor) RNA-seq datasets were employed for this purpose. We conducted weighted gene co-expression network analysis (WGCNA) to identify key modules and hub genes associated with melanoma. Log-rank analysis and multivariate Cox model analysis were performed to identify prognosis genes, which were validated using two independent melanoma datasets. We also evaluated the correlation between prognostic gene and immune cell infiltration.

**Results:**

The blue module was the most relevant for melanoma and was thus considered the key module. Intersecting genes were identified between this module and differentially expressed genes (DEGs). Finally, 72 genes were identified and verified as hub genes using the Oncomine database. Log-rank analysis and multivariate Cox model analysis identified 13 genes that were associated with the prognosis of the metastatic melanoma group, and *RTP4* was validated as a prognostic gene using two independent melanoma datasets. *RTP4* was not previously associated with melanoma. When we evaluated the correlation between prognostic gene and immune cell infiltration, we discovered that *RTP4* was associated with immune cell infiltration. Further, *RTP4* was significantly associated with genes encoding components of immune checkpoints (*PDCD1*, *TIM-3,* and *LAG3*).

**Conclusions:**

RTP4 is a novel prognosis-related hub gene in cutaneous melanoma. The novel gene *RTP4* identified here will facilitate the exploration of the molecular mechanism of the pathogenesis and progression of melanoma and the discovery of potential new target for drug therapy.

**Supplementary Information:**

The online version contains supplementary material available at 10.1186/s41065-021-00183-z.

## Introduction

Melanoma and nonmelanoma represent the two main types of skin cancer. The most common nonmelanomas are basal cell and squamous cell carcinomas. Nonmelanoma skin cancers are the most frequently occurring skin cancers, with greater than one million diagnoses worldwide in 2018. Melanoma represents 21.6% of skin cancer cases. According to the GLOBOCAN database (gco.iarc.fr), in 2018, there were 287,723 new cases of melanoma worldwide. Melanoma is more aggressive and has a high mortality rate compared with other types of skin cancers, and its mortality rate is approximately 8-times higher compared with that of nonmelanoma skin cancer [1, 2]. The poor prognosis of patients with melanoma is associated with its high metastatic potential [3]. The main causes of death include extensive metastasis to the lung, liver, bone, and brain [4]. Early diagnosis and treatment of melanoma are therefore required to reduce its significant threat to human health.

Hyperplasia of abnormal melanocytes is a risk factor for melanoma, although most such hyperplasias are benign. However, some are associated with a significant risk of developing into a melanoma or already have developed its malignant phenotype. Among numerous studies aimed to identify the mechanism of melanoma, one study identified differentially expressed genes (DEGs) between metastatic and primary melanomas. These DEGs were enriched in mRNAs encoding cell adhesion and proliferation molecules. A protein–protein interaction network was also constructed, and some key genes with higher degrees in the network has been identified, such as *PCNA*, *CDK1*, and *MAD2L1* [5]. As disruption of the epigenomic landscape is recognized as a widespread feature inherent in tumor development and progression, a study of DNA methylation identified markers associated with the melanoma. These identified methylation biomarkers involved in melanoma development (e.g., *HOXA9* methylation) and tumor progression (e.g., *TBC1D16* methylation). In addition, they determined *PON3* DNA methylation as biomarkers with prognostic information independent of tumor thickness and ulceration [6]. Mutational analysis of melanoma tissue was another strategy to identify biomarkers between metastatic and primary melanomas. Numerous genes that have somatic mutations were identified, such as *BRAF* and *TERT* [7].

Weighted gene co-expression network analysis (WGCNA) is a systematic biological approach for constructing weighted correlation networks to identify key modules that are highly associated with clinical traits. Moreover, WGCNA is used to measure relationships between modules and genes as well as to rank genes in modules of interest with clinical data [8]. WGCNA can identify core-related genes, which may be involved in important pathological processes and have important clinical application. WGCNA is therefore widely used to conduct association analyses of gene sets with diseases and to identify candidate hub genes, particularly of patients with cancer [9, 10]. For example, a WGCNA study of pancreatic ductal adenocarcinoma identified five modules and found 10 hub genes that may indicate poor prognosis [11]. It has been established that the metastatic ability of melanoma is regulated by an intricate gene network. Thus, WGCNA method was also applied to investigate the relationship between the key module and hub genes associated with the metastasis ability of melanoma. *PKP1* was identified as a new potential tumor suppressor in human melanoma, likely through regulating calcium signaling pathways [12]. Another study screened out *SMARCA4* associated with metastasis melanoma, which in turn affects the signal transduction of the adherens junction [13]. Other genes *CCNB2*, *ARHGAP30*, and *SEMA4D* were also identified by WGCNA, which associated with survival as potential prognostic predictors and molecular targets of treatment [14].

Biomarkers for prognosis of cutaneous melanoma have drawn intensive attentions in recent years, and many studies have identified plenty of effective biomarkers and therapeutic targets in metastatic melanoma. To improve our understanding of the biological pathology of metastatic melanoma, we applied WGCNA to identify key modules and hub genes associated with melanoma. Prognostic genes were identified using multivariate Cox model analysis. We further examined the associations between prognostic genes, immune cell infiltration, and immune checkpoints.

### Materials and methods

#### Data collection

GTEx (normal skin) and TCGA (melanoma tumor, SKCM) total gene RNA-seq datasets were obtained from UCSC Xena (https://xenabrowser.net/). TOIL was used to reprocess raw GTEx and TCGA counts data to correct for batch effects and to conduct merge-analysis of GTEx and TCGA datasets [15]. The combined data set included 469 melanoma samples and 812 normal samples. Basic clinical information of the 469 melanoma samples was downloaded from the UCSC Xena website, including sample type, Breslow depth, vital status, sex, ulceration, survival time, and TNM stage (Table S1). The samples comprised 367 metastatic and 102 primary melanomas, respectively.

### Analyses of DEGs

mRNA expression data were extracted from the total gene expression data, yielding 19,521 mRNA genes for analysis. The DEGs were identified using the DESeq2 software package [16]. In this study, a |log2-fold change (log2FC)|> 2 and adjusted *P* < 0.01 were selected as the standard cutoffs to identify DEGs. Gene expression levels of normal skin and all tumor samples were determined, including comparisons of those of normal skin and all tumors, normal skin and primary tumors, normal skin and metastatic tumors, and primary and metastatic tumors.

#### WGCNA

We used the WGCNA package to construct gene co-expression networks. Raw mRNA gene counts were normalized to FPKM values, then converted to log2 (FPKM + 1) values. First, we excluded genes that were not detected using the expression profile and then calculated the variance of each included gene. Genes with standard deviations in the top 20% were subjected to further analysis. Certain samples were distant, and outliers were excluded according to their cluster distances.

To construct a weighted gene network, the soft threshold power β was defined as 6, which was the lowest power based on a scale-free topology [17]. After constructing a scale-free network, the expression matrix was transformed into an adjacency matrix and then transformed into a topological matrix. We used the average-linkage hierarchical clustering method, according to the topological overlap measure (TOM), to cluster genes, and we accordingly defined the minimum number of each gene network module as 30. The threshold for similar module combinations was defined as 0.25. Therefore, after identifying gene modules using dynamic shear, we calculated module eigengenes (MEs, the first principal component of one module), clustered the modules, and merged closer modules into new modules according to height = 0.25. To identify modules that were significantly associated with clinical traits, we generated a heat map of module-trait relationships according to the tutorial included in the WGCNA package of R. Genes in the most significant module were selected for subsequent analysis.

### PPI network construction

Overlapping genes between significant modules and DEGs were selected for protein–protein interaction (PPI) analysis. The STRING tool provides information for this purpose [18]. PPI analysis may reveal protein function and identify cellular mechanisms through protein interactions. Here we established a PPI network of potential genes, and genes not significantly associated with other genes in the PPI network were excluded from subsequent analyses.

### Functional enrichment analysis of network module genes

To analyze the functions of genes in the modules, we conducted Gene Ontology (GO) [19] and Kyoto Encyclopedia Gene and Genomes (KEGG pathway) [20] analyses using the cluster-Profiler package [21]. We identified overrepresented GO terms in the categories as follows: biological process (BP), molecular function (MF), and cellular component (CC). Those in KEGG pathway were similarly identified. The false discovery rate (FDR) *P* value < 0.05 was selected as the threshold.

### Identification and validation of hub genes

Hub genes are highly connected within a module and are significantly associated with biological functions [22]. Here we defined hub genes with high module membership (MM) (|cor.weighted|> 0.85). We used the Oncomine database (https://www.oncomine.org) to verify the expression of hub genes, which included the melanoma datasets as follows: haqq, riker, and talantov. A meta-analysis was performed that employed analysis of *P* values to evaluate the significance of differences in gene expression between melanomas and normal skin. *P* < 0.05 represents significant difference.

### Identification and validation of prognostic genes

Prognostic genes were screened according to prognostic information into metastatic melanoma and primary melanoma groups. Patients were stratified into a high-level group or a low-level group according to the median expression level, and the Kaplan–Meier method was used to analyze survival. The log-rank test was used to compare the survival curves of patients in different groups, and *P* < 0.05 indicates significant differences. Analysis of a Cox proportional hazards model was performed after adjusting for sex, age upon diagnosis, and tumor stage. *P* < 0.05 represents a significant difference. The GSE65904 [23] and GSE22153 [24] of the GEO database were used for validating potential prognostic genes. The GSE65904 and GSE22153 datasets included 188 and 57 metastatic melanoma samples, respectively. Analysis using a Cox proportional hazards model was performed after adjusting for sex and age upon diagnosis. *P* < 0.05 indicates a significant difference.

### Immune-related analysis

The microenvironment of melanoma tissue comprises tumor and immune cells as well as their secreted molecules such as proinflammatory and anti-inflammatory factors, which ultimately determine the malignant phenotype of the tumor. We therefore used the Tumor Immune Estimation Resource (TIMER) tool (http://cistrome.org/TIMER/) to evaluate the correlation between prognostic genes and immune cell infiltration in SKCM-Metastasis, including B cells, CD8 + T cells, CD4 + T cells, macrophages, neutrophils, and dendritic cells (DCs) [25]. Purity-corrected partial Spearman’s rho value and statistical significance were applied. For gene expression levels compared with tumor-cell purity, *P* < 0.05 indicates a significant difference. Further, gene expression data were evaluated for immune checkpoint genes, including *PDCD1* (PD1), *CD274* (PD-L1), *PDCD1LG2* (PD-L2), *CTLA4*, *TIM-3*, and *LAG3*. |log2FC)|> 1 and *P* < 0.01 were considered significant. The relationship between prognostic genes and immune checkpoints such as *PDCD1*, *CD274*, *PDCD1LG2*, *CTLA4*, *TIM-3*, and *LAG3* were evaluated using Pearson’s correlation analysis.

### Druggable targets

The ChEMBL and Drugbank databases comprise pharmacodynamic and pharmacokinetic information for bioactive compounds and drugs [26]. We searched for immune-related genes among the two databases to identify genes as targets of approved drugs or bioactive compounds.

### Results

#### Detection of DEGs

**Fig. 1:**
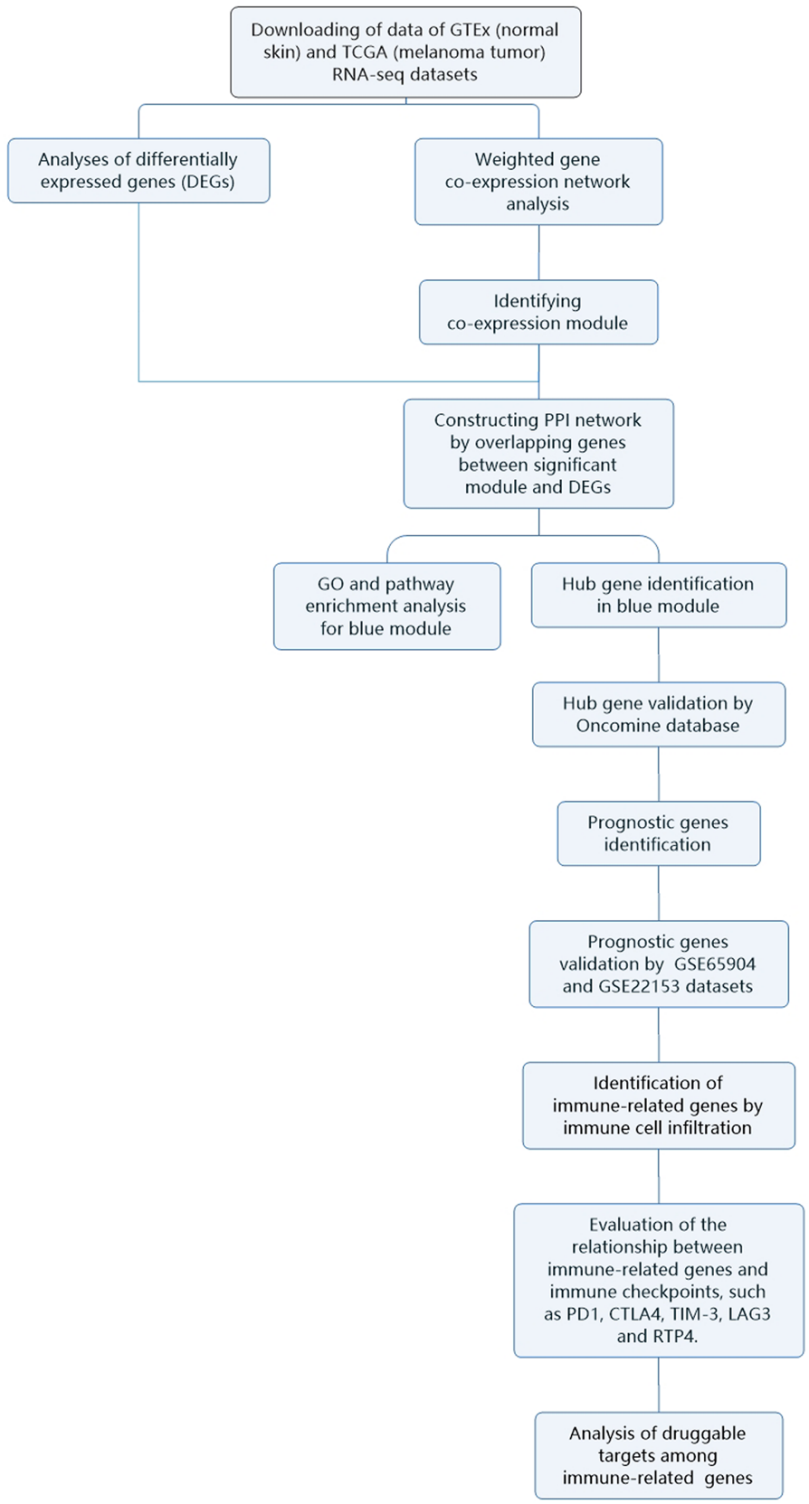
Analytical procedures

**Fig. 2:**
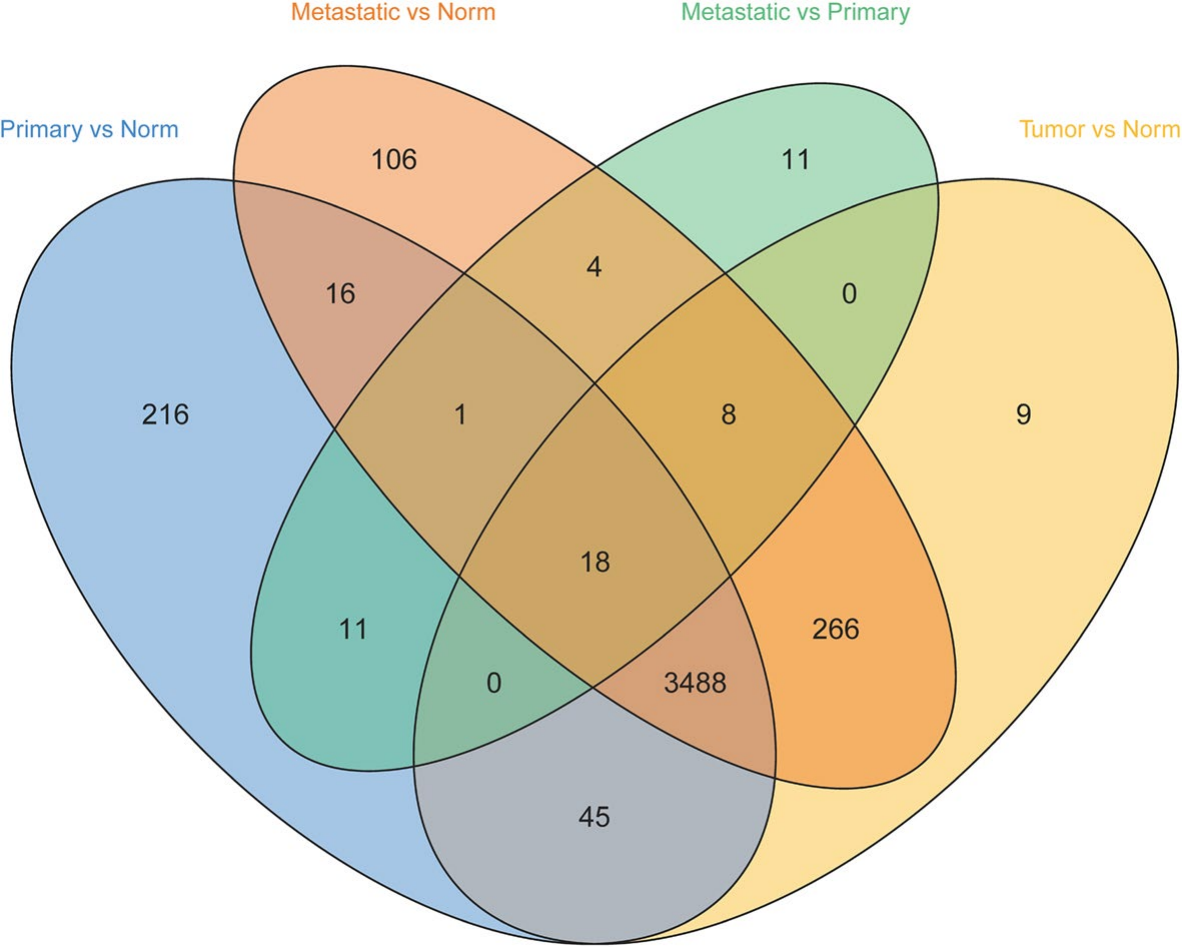
Common differentially expressed genes. A Venn diagram was utilized to screen the common genes. The comparison was made between normal skin and all tumor samples. Further comparisons included normal skin and primary tumors, normal skins and metastatic tumors, and primary and metastatic tumors

The procedures used in our study were illustrated in the flow chart in Fig. 1. The gene expression levels of normal skin and tumor samples were compared. We detected 3,834 DEGs. Compared with 812 normal samples, 2,212 downregulated genes and 1,622 upregulated genes were identified in the 469 melanoma samples (Fig. S1). We further compared normal skin and primary tumors, normal skin and metastatic tumors, and primary tumors and metastatic tumors (Fig. 2). We identified 18 significant DEGs among all comparisons, indicating that they may play continuous roles in tumor development (Table S2), including *AGR3*, *CRP*, *HTN3*, *KRT26*, *KRT38*, *KRTAP10-3*, *KRTAP10-4*, *KRTAP10-5*, *KRTAP10-7*, *KRTAP10-8*, *KRTAP10-9*, *KRTAP1-1*, *KRTAP2-4*, *KRTAP4-5*, *PRH2*, *SAGE1*, *TBC1D3*, and *TKTL1*. Among these genes, 11 encode keratins or related proteins. We further identified 11 DEGs in a comparison of metastatic tumors with primary tumors (Table S3), which were not detected in the comparisons between other groups, including *HMX1*, *ALB*, *ORM2*, *PRB3*, *SFTA3*, *RTL1*, *CRABP1*, *OR1E1*, *APOH*, *FBN3*, and *IGFL1*. These results indicate that these genes play an important role in tumor metastasis.

### A weighted gene co-expression network

**Fig. 3:**
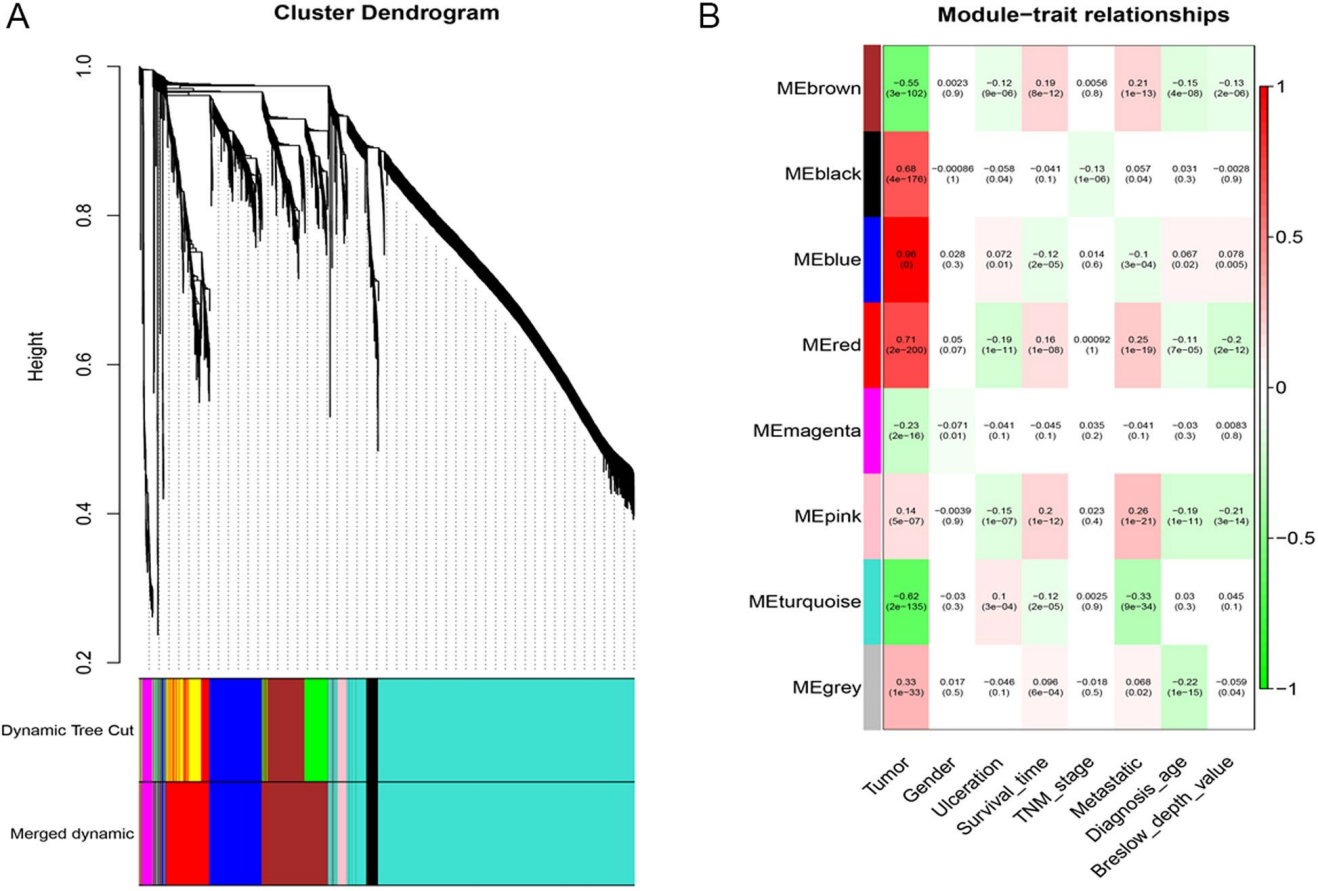
Identification of a module associated with clinical features. **a** Dendrogram of expressed genes clustered according to a dissimilarity measure (1-TOM). Dynamic Tree Cut corresponds to the original module, and Merged Dynamic corresponds to the final module. **b** Heat map of the correlation between modular significant and clinical features

We defined β = 6 (scale-free R^2^ = 0.95) as the appropriate soft-thresholding value to satisfy the scale-free network criteria. We identified eight modules, which were assigned different colors (Fig. 3a). We calculated the correlation between modules and clinical traits (Fig. 3b). These analyses show that tumor (melanoma) significantly correlated with most of the modules in the eight clinical traits. According to the correlation between MEs and traits, the blue module (n = 438 genes) was the most relevant, and therefore, a key module associated with melanoma.

### PPI network construction

There were 350 intersecting genes between the blue module and DEGs, which were used to constructed a PPI network for validating gene co-expression network proteins. A node represents a gene in the network, and the edge represents the interaction between genes. Genes not linked to other genes in the PPI network were deleted, and a PPI network was reconstructed using the remaining genes. The network contained 264 nodes and 716 edges (Fig. S2). The PPI data indicated that these genes had a significant regulatory relationship and that 264 genes of the blue module may interact.

### GO and pathway enrichment analysis

**Fig. 4:**
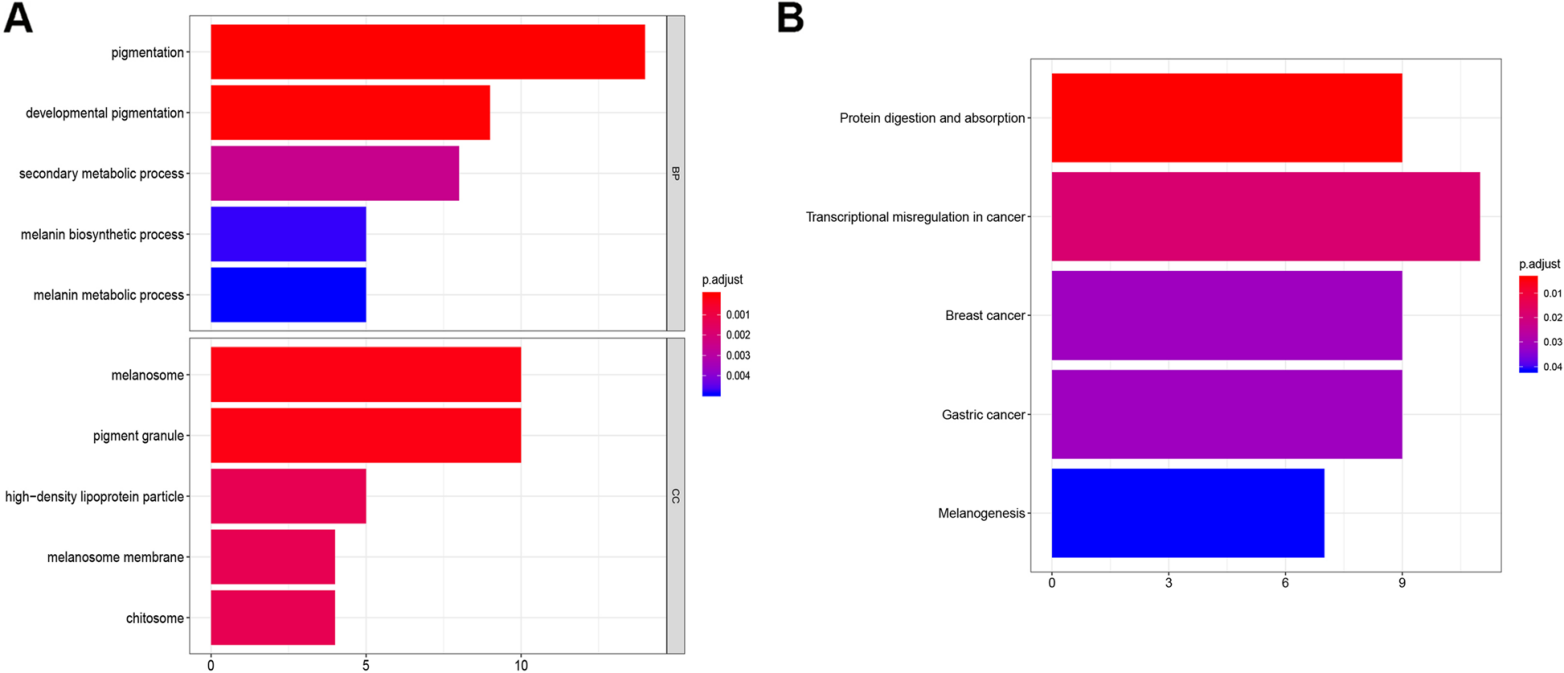
Functional enrichment of genes in the blue module. **a** Gene Ontology (GO) functional enrichment of genes in the blue module. The x-axis represents the number of genes of each term and the y-axis shows the GO terms. BP: biological process, CC: cell component. **b** Kyoto Encyclopedia Gene and Genomes (KEGG) functional enrichment of genes in the blue module. The x-axis represents the number of genes of each term and the y-axis shows the KEGG terms

The genes in the blue module were selected for GO and KEGG pathway enrichment analysis. The most overrepresented GO terms in BP were associated with melanin, including pigmentation, melanin biosynthesis, and metabolism as well as developmental pigmentation. In the CC categories, the enriched GO terms in the blue module were primarily associated with melanosome and pigment granule (Fig. 4a). According to the KEGG database, the genes in the blue module were mainly enriched for transcriptional dysregulation in cancer, protein digestion and absorption, breast cancer, gastric cancer, and melanogenesis pathways (Fig. 4b).

### Hub gene identification and validation

The screening criteria of hub genes in the key module (|MM|> 0.85), identified 85 genes in the blue module as hub genes in the co-expression network. Meta-analysis of the Oncomine database employed the *P* value to validate expression differences between hub genes of melanoma and normal skin. Seventy-two genes were validated (*P* < 0.05) in this module. We generated a heat map to visualize the expressing of these 72 hub genes in DEG- discovery stage **(**Fig. S3).

### Identification and validation of prognostic genes

**Fig. 5:**
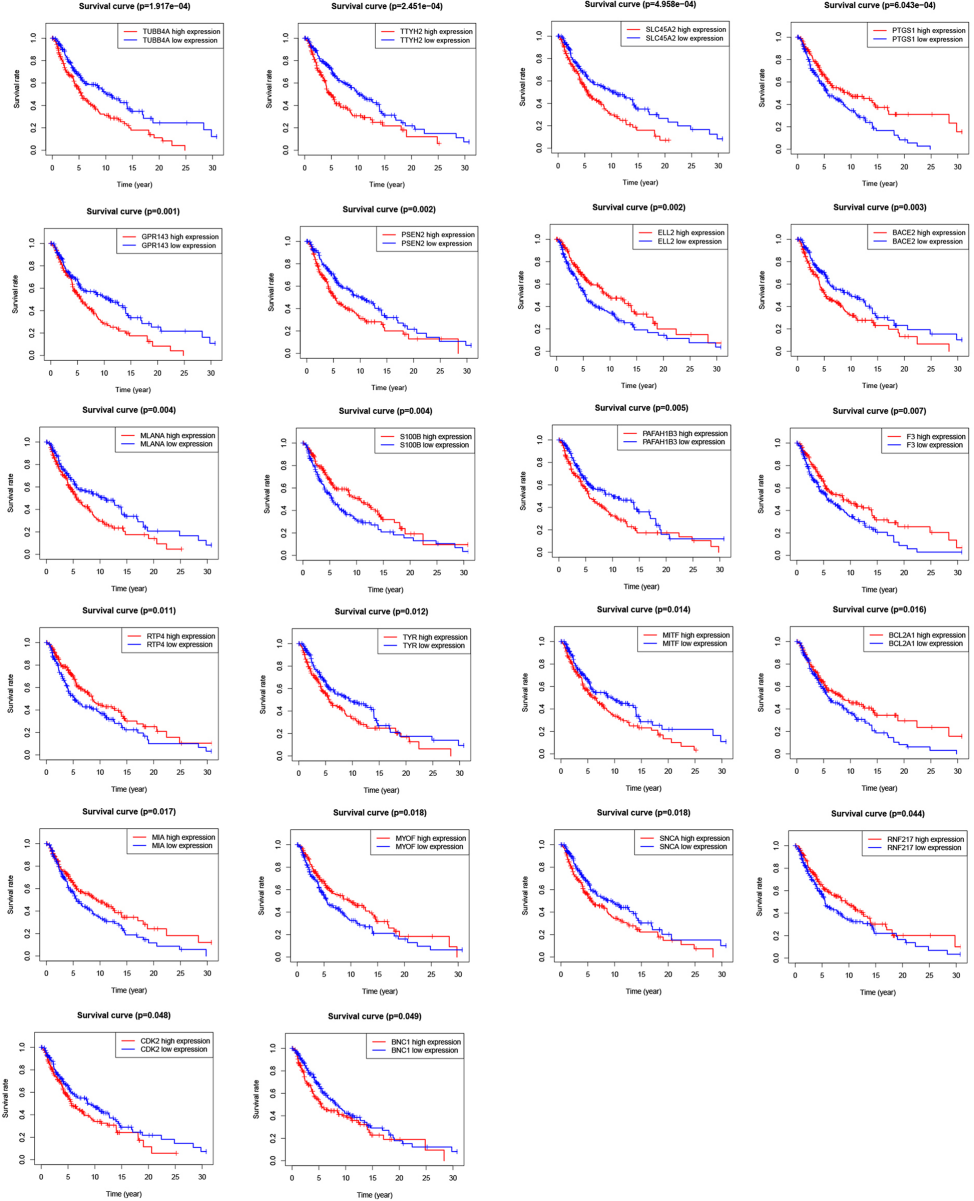
Overall survival associated with the prognostic genes expressed by patients with metastatic melanoma. Patients were stratified into a high-level or low-level group according to median expression levels

We used log-rank analysis to evaluate the difference in overall survival between high and low expression levels of the 72 hub genes. The prognostic genes screened according to prognostic information were allocated into separate groups of metastatic melanoma or primary melanoma. This procedure found that 22 genes were significantly associated with the prognosis of patients in the metastatic group (Fig. 5), although a prognosis-associated gene was not identified in the primary group. Multivariate analysis revealed that 13 genes remained associated with prognosis (Table 1). Analysis of two independent datasets (GSE65904 and GSE22153) validated that *RTP4* was a significant prognostic gene (Tables S4 and Table S5). Above results demonstrated that patients with metastatic melanoma with high expression of *RTP4* experienced significantly longer overall survival than those with low expression. The *RTP4* gene can act as an independent prognostic factor independent of other clinical traits.

**Table 1:** Prognostic genes in patients with metastatic melanoma identified using a multivariate Cox model

Gene	HR	HR.95L	HR.95H	P value
PTGS1	0.7006072	0.5846867	0.8395102	0.0001154
MYOF	0.7953699	0.6984751	0.9057063	0.0005519
RTP4	0.8082045	0.710303	0.9196	0.0012284
ELL2	0.7221818	0.5909052	0.8826232	0.001474
TUBB4A	1.123089	1.0383324	1.2147641	0.0037371
TTYH2	1.1823222	1.0523902	1.328296	0.0048074
S100B	0.9110047	0.8518034	0.9743206	0.0065519
GPR143	1.0875581	1.020966	1.1584936	0.0092254
CDK2	1.1424483	1.0327182	1.2638376	0.0097424
SLC45A2	1.0860675	1.0185165	1.1580987	0.0117374
BCL2A1	0.9039319	0.8260128	0.9892012	0.0280894
PAFAH1B3	1.2696524	1.0183585	1.5829565	0.0338688
MIA	0.9370072	0.8794632	0.9983163	0.044212

*Abbreviations*: *HR* hazard ratio; Analysis of a Cox proportional hazards model was performed after adjusting for sex, age upon diagnosis, and tumor stage

### Immune-related genes

**Fig. 6:**
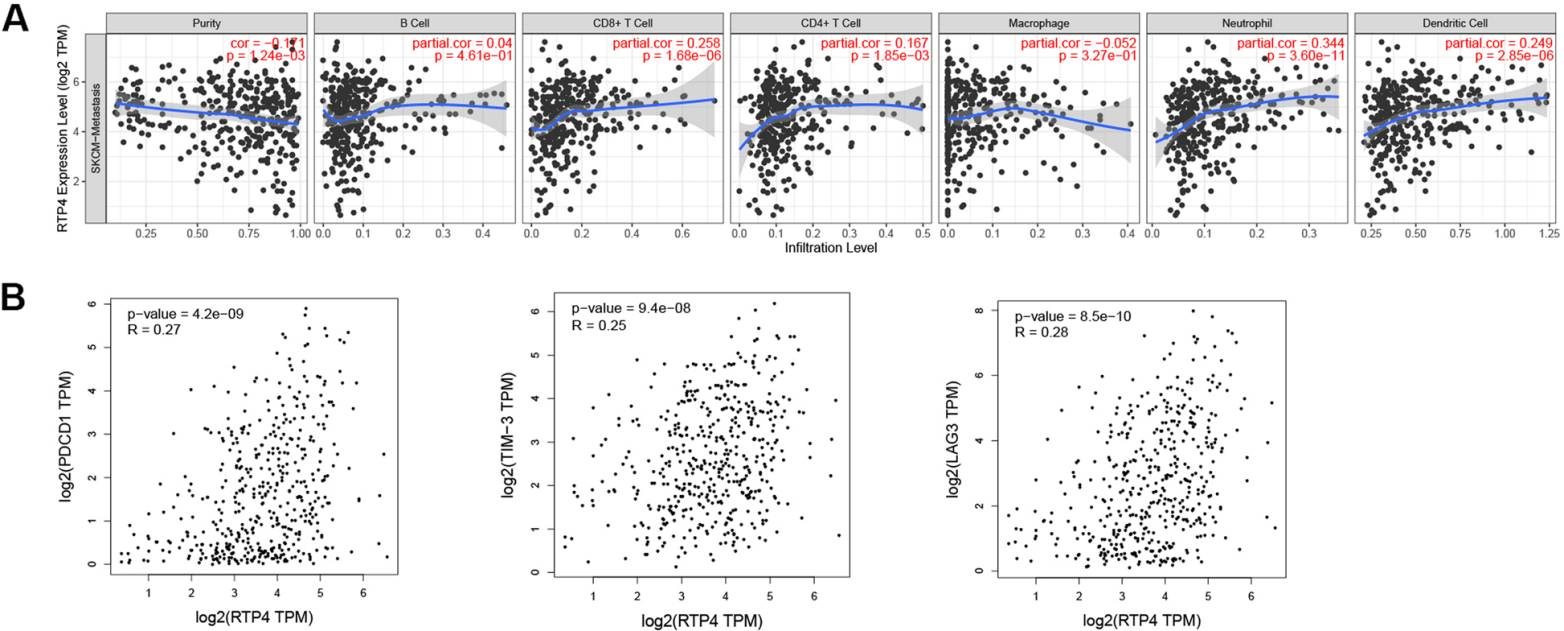
Effect of *RTP4* on immune cell infiltration and immune checkpoint genes. **a** The correlation between *RTP4* and immune cell infiltration. **b** Correlation between immune checkpoint genes and *RTP4*

Immune cell infiltration is highly associated with tumor development. Therefore, TIMER was utilized to examine the correlations between the expression levels of prognostic genes and immune cell infiltration. We discovered that *RTP4* was associated with immune cell infiltration of samples of melanoma metastases, which negatively correlated with the cellular purity of melanomas and positively correlated with neutrophils (Fig. 6a). Gene expression levels were evaluated to identify immune checkpoint genes, including *PDCD1*, *CD274*, *PDCD1LG2*, *CTLA4*, *TIM-3*, and *LAG3*. *PDCD1*, *CTLA4*, *TIM-3*, and *LAG3* were upregulated in melanomas compared with normal skin. Subsequently, correlations between immune checkpoints (*PDCD1*, *CTLA4*, *TIM-3*, *LAG3*) and *RTP4* were evaluated. We found that *RTP4* positively correlated with the genes mentioned above, including *PDCD1* (r = 0.27, *P* = 4.2E-9), *TIM-3* (r = 0.25, *P* = 9.4E-8), and *LAG3* (r = 0.28, *P* = 8.5E-10) (Fig. 6b).

### Druggable targets

To determine whether the immune-related gene *RTP4* expressed by patients with melanoma had predicted drug targets, we queried the ChEMBL and Drugbank databases. We were unable to identify such *RTP4* targets. More efforts need to be made for drug development targeting this gene.

### Discussion

The prognosis of patients with melanoma predominantly depends on the disease stage. The prognosis of patients with local disease is generally favorable, and the 5-year survival rate exceeds 90% [27]. Therefore, early diagnosis is critically important for administering effective treatment. The identification of diagnostic or prognostic biomarkers is therefore increasingly important. To this end, here we screened for potential biomarkers associated with melanoma using TCGA data. When we used WGCNA to identify gene modules significantly associated with melanoma, we identified a module (blue) as that most relevant to melanoma. This module comprised 85 genes with a correlation > 0.85. After validation using the Oncomine database, 72 hub genes were validated. The prognostic value of these biomarkers was evaluated using the information included in this dataset. Among them, 13 genes were significantly associated with the prognosis of patients assigned to the metastatic group. Two datasets were used to validate prognostic significance, and *RTP4* was validated.

*RTP4* was identified and validated as a prognostic gene. It is a novel gene associated with melanoma, which was not discovered in other melanoma studies. *RTP4* has the potential to be an effective biomarkers and therapeutic targets in metastatic melanoma. *RTP4* encodes a receptor transporter protein, which facilitates trafficking and functional cell surface expression of certain G protein-coupled receptors (GPCRs). *RTP4* represents a novel targeting opportunity for many kinds of cancers. For example, two studies link *RTP4* with prostate and breast cancer, respectively. One study found that *RTP4* is a novel methylated biomarker for accurate diagnosis and treatment of prostate adenocarcinoma [28]. Another study discovered that the expression level of *RTP4* independently predicts breast cancer outcomes of HER2( +) patients [29]. Further, *RTP4* is required for antigen-dependent immune editing of cancer cells using CRISPR screens [30]. *RTP4* plays a role in diverse viral infections such as those caused by HPV16 and influenza virus [31, 32]. Canonical interferons (IFNs) induce interferon-stimulated genes to exert their antiviral activities. *RTP4* is induced by type I IFN (IFN-I) and binds the TANK-binding kinase (TBK1) complex to negatively regulate TBK1 signaling through interference with the expression and phosphorylation of TBK1 [33].

Immune cell infiltration is highly associated with the development of the tumor, and we therefore evaluated the correlation between prognostic gene expression and immune cell infiltration. As a result, we discovered that *RTP4* was associated with immune cell infiltration of melanoma metastases. *RTP4* expression negatively correlated with melanoma purity and highly correlated with neutrophils. Many types of immune cells migrate close to tumors and exert an anti-tumor effect [34]. Further, these infiltrating immune cells can induce tumor cells to produce immunosuppressive phenotypes, thereby promoting tumor growth. Thus, the role of neutrophils in tumors has attracted our attention. The close correlation between neutrophils and *RTP4* indicates that *RTP4* contributes to the regulation of neutrophil function. We further discovered that *PDCD1*, *CTLA4*, *TIM-3*, *LAG3*, and *RTP4* were upregulated in melanomas. *RTP4* was positively correlated with the genes mentioned above, including *PDCD1*, *TIM-3*, and *LAG3*, suggesting that immune checkpoint inhibitors may be considered for treating such patients.

Gene expression comparisons were made between normal skin and all tumor samples, normal skin and primary tumors, normal skin and metastatic tumors, and primary and metastatic tumors. Eighteen genes were significantly differentially expressed in all comparisons, indicating that these genes may play a continuous role in melanoma development. After reviewing the literature, no study on the relationship between these genes and melanoma was found; and the roles of these genes in melanoma therefore deserve further study. Among these genes, 11 encode keratins or related proteins. *KRT26* and *KRT38* are members of the keratin gene family and belong to type I keratins. Diseases associated with *KRT38* include nodular basal cell carcinoma [35]. *KRTAP10-3*, *KRTAP10-4*, *KRTAP10-5*, *KRTAP10-7*, *KRTAP10-8*, *KRTAP10-9*, *KRTAP1-1*, *KRTAP2-4*, and *KRTAP4-5* encode keratin-associated proteins, which are members of the keratin-associated protein (KAP) family. The KAP proteins form a matrix of keratin intermediate filaments, which contribute to the structure of hair fibers. Eleven genes were significantly differentially expressed between metastases and primary tumors, and *ALB*, *ORM2*, *CRABP1*, and *APOH* may be associated with the metastasis of melanoma. These genes are associated with several cancers, such as *ALB* that is associated with lymph node metastasis in lung squamous cell carcinoma [36]. The C-reactive protein (CRP)/albumin (ALB) ratio serves as a prognostic marker for several cancers [37]. There is no relevant functional study on the relationship between *ALB*, *ORM2*, *CRABP1*, and *APOH* and melanoma metastasis, which may represent future candidates.

## Conclusions

Our aim here was to identify gene networks and hub genes associated with melanoma and to further identify the underlying mechanisms responsible for oncogenesis and tumor progression, including metastasis. WGCNA and survival analysis were used to identify key hub genes with prognostic value for melanoma. These identified prognostic genes were validated by two independent metastasis melanoma datasets. We also evaluated the correlation between prognostic gene and immune cell infiltration. *RTP4* was significantly associated with the prognosis of patients with melanoma and was defined as a prognostic gene. We further discovered that *RTP4* was associated with immune cell infiltration, which negatively correlated with the cellular purity of melanomas and positively correlated with neutrophils. Further, *RTP4* was significantly associated with genes encoding components of immune checkpoints (*PDCD1*, *TIM-3*, and *LAG3*). Notably, *RTP4* is a novel gene not previously associated with melanoma. Our research therefore provides more information for exploring the mechanisms responsible for the development of melanoma.

## Supplementary Information

**Additional file 1: Figure S1.** Volcano plots reflecting significant differentially expressed genes by patients with melanoma compared with normal controls. **Figure S2.** Protein-protein interaction network of genes in the blue module. **Figure S3.** Heat map of 72 validated hub genes during the discovery stage of differentially expressed genes

**Additional file 2: Table S1.** Basic clinical information of 469 melanoma samples. **Table S2.** Common differentially expressed genes in all comparisons. The gene expression comparison was made between normal skin and all tumor samples,normal skin and primary tumors, normal skin and metastatic tumors, and primary tumors and metastatic tumors. **Table S3.** Eleven genes were significantly differentially expressed in the comparison of metastatic tumors with primary tumors. **Table S4.** Prognostic genes in patients with metastatic melanoma identified using the GSE65904 dataset. **Table S5.** Prognostic genes in patients with metastatic melanoma identified using the GSE22153 dataset

## Data Availability

The datasets supporting the conclusions of this article are included within the article. Materials are available from the corresponding author on reasonable request.

## Abbreviations

TCGA: The Cancer Genome Atlas
WGCNA: Weighted gene co-expression network analysis
DEGs: Differentially expressed genes
GO: Gene Ontology
KEGG: Kyoto Encyclopedia Gene and Genomes
BP: Biological process
MF: Molecular function
CC: Cellular component
FDR: False discovery rate
PPI: Protein–protein interaction
