# Supplementary material for: RTP4 is a novel prognosis-related hub gene in cutaneous melanoma
Source: Hereditas. 2021 Jun 21;158:22. doi: 10.1186/s41065-021-00183-z (PMC8215788; doi:10.1186/s41065-021-00183-z)

**Fig. S1** Volcano plots reflecting significant differentially expressed genes by patients with melanoma compared with normal controls

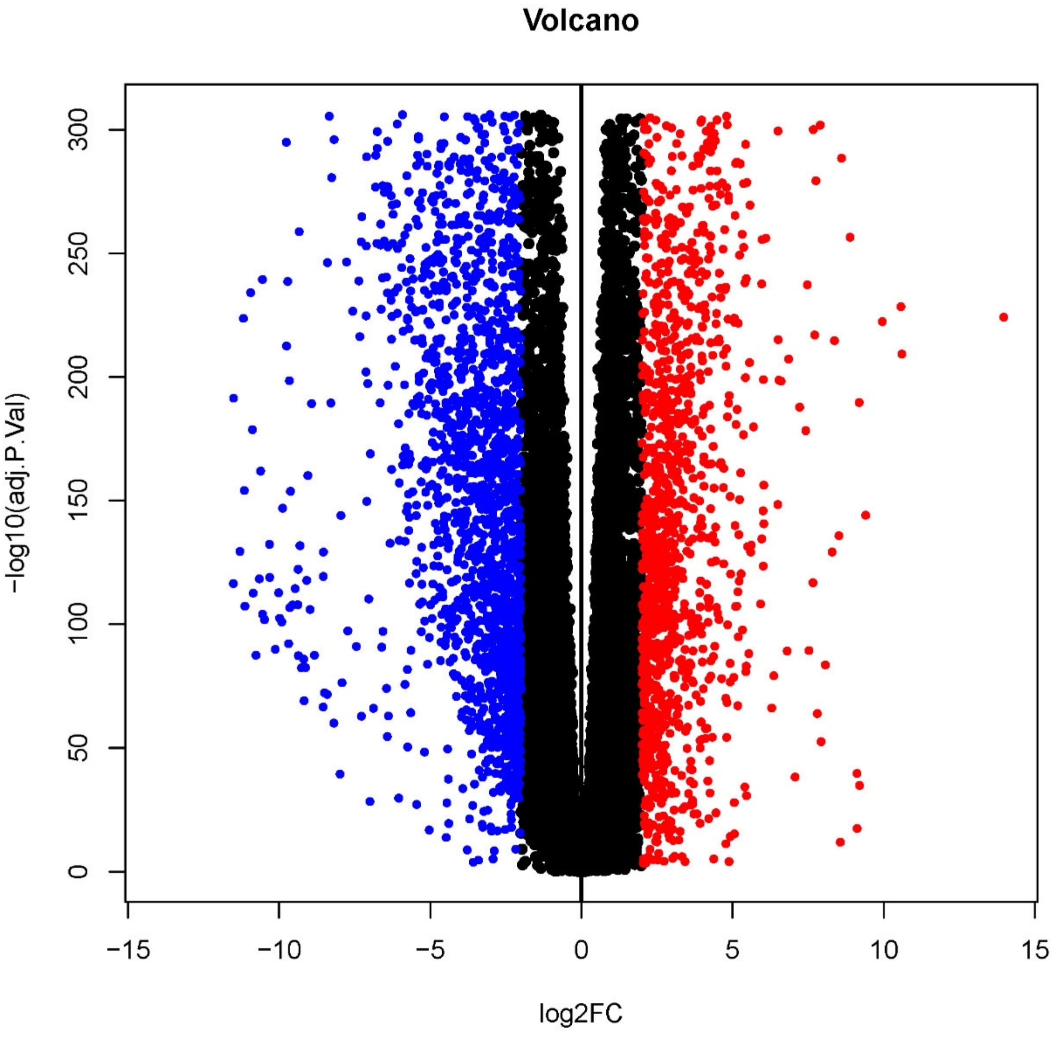

**Fig. S2** Protein-protein interaction network of genes in the blue module

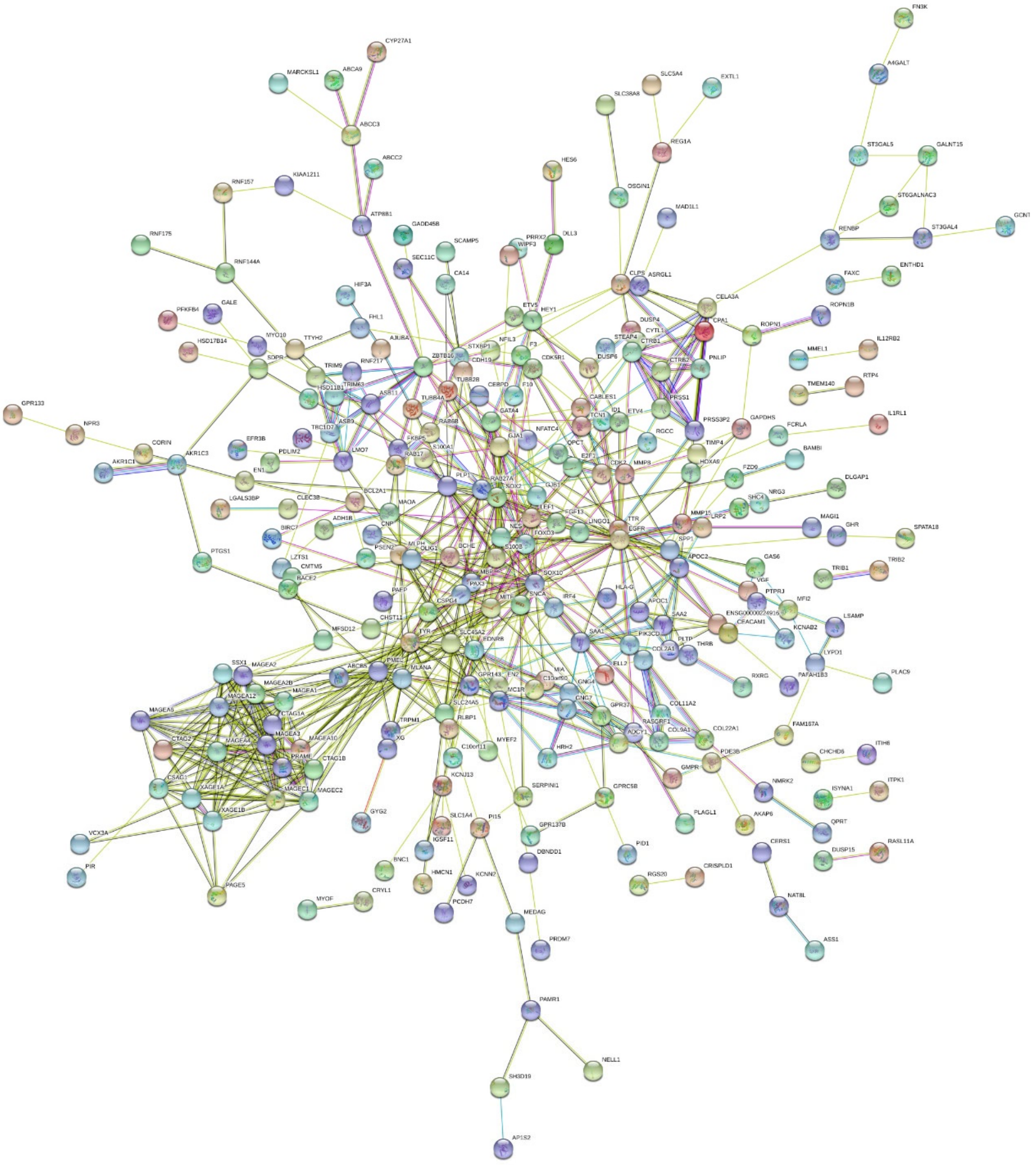

**Fig. S3** Heat map of 72 validated hub genes during the discovery stage of differentially expressed genes

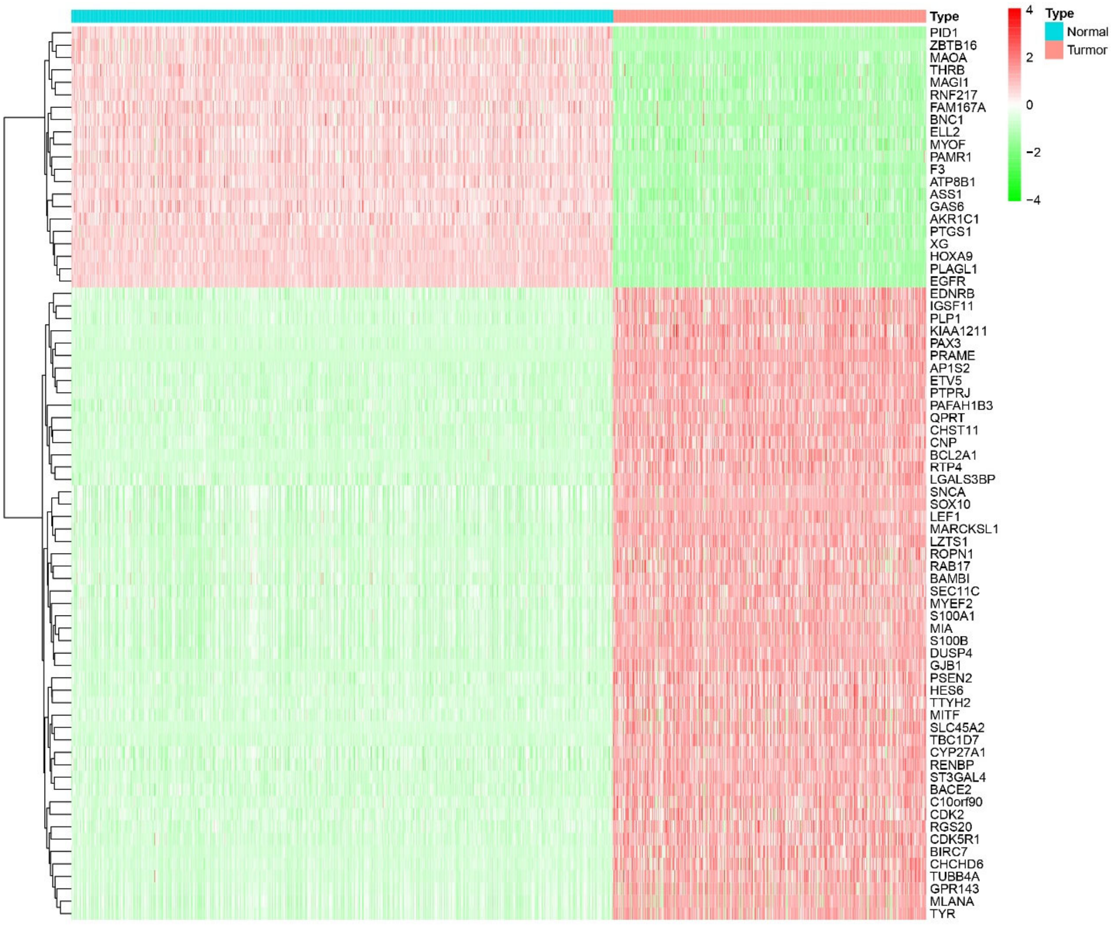

Supplement: Supplementary file 1 — Additional file 1: Figure S1. Volcano plots reflecting significant differentially expressed genes by patients with melanoma compared with normal controls. Figure S2. Protein-protein interaction network of genes in the blue module. Figure S3. Heat map of 72 validated hub genes during the discovery stage of differentially expressed genes [file 41065_2021_183_MOESM1_ESM.pdf]
