# Supplementary material for: RTP4 is a novel prognosis-related hub gene in cutaneous melanoma
Source: Hereditas. 2021 Jun 21;158:22. doi: 10.1186/s41065-021-00183-z (PMC8215788; doi:10.1186/s41065-021-00183-z)
Supplement: Supplementary file 2 — Additional file 2: Table S1. Basic clinical information of 469 melanoma samples. Table S2. Common differentially expressed genes in all comparisons. The gene expression comparison was made between normal skin and all tumor samples,normal skin and primary tumors, normal skin and metastatic tumors, and primary tumors and metastatic tumors. Table S3. Eleven genes were significantly differentially expressed in the comparison of metastatic tumors with primary tumors. Table S4. Prognostic genes in patients with metastatic melanoma identified using the GSE65904 dataset. Table S5. Prognostic genes in patients with metastatic melanoma identified using the GSE22153 dataset [file 41065_2021_183_MOESM2_ESM.pdf]

Table S1 Basic clinical information of 469 melanoma samples

Samples	Sample_type	Vital_status	Survival_time	Gender	TNM_stage	Diagnosis_age	Breslow_depth_value	Ulceration
TCGA-D3-A8GN-06	Metastatic	Alive	4897	female	i/ii nos	27	NA	NA
TCGA-D3-A2JL-06	Metastatic	Alive	5219	female	i/ii nos	43	NA	NA
TCGA-D3-A51E-06	Metastatic	Alive	5318	female	i/ii nos	39	1.2	NA
TCGA-FR-A3YO-06	Metastatic	Alive	NA	female	i/ii nos	NA	NA	NA
TCGA-D3-A3C3-06	Metastatic	Alive	NA	female	i/ii nos	NA	NA	NA
TCGA-XV-A9W5-01	Primary Tumor	Alive	392	male	i/ii nos	51	2	NA
TCGA-D3-A3CB-06	Metastatic	Alive	5065	male	i/ii nos	39	1.75	NA
TCGA-D3-A3C1-06	Metastatic	Alive	NA	male	i/ii nos	NA	NA	NA
TCGA-D3-A8GO-06	Metastatic	Dead	NA	female	i/ii nos	NA	1.2	NA
TCGA-EE-A29N-06	Metastatic	Dead	566	male	i/ii nos	78	NA	NA
TCGA-D3-A1Q5-06	Metastatic	Dead	3424	male	i/ii nos	60	NA	NA
TCGA-FS-A1ZQ-06	Metastatic	Dead	4062	male	i/ii nos	31	NA	NA
TCGA-D3-A8GV-06	Metastatic	Dead	5101	male	i/ii nos	25	NA	NA
TCGA-FS-A1ZC-06	Metastatic	Dead	10870	male	i/ii nos	51	NA	NA
TCGA-RP-A690-06	Metastatic	Alive	6	female	NA	66	NA	NA
TCGA-HR-A2OG-06	Metastatic	Alive	7	female	NA	50	NA	NA
TCGA-D9-A1JX-06	Metastatic	Dead	216	female	NA	80	NA	NO
TCGA-GN-A266-06	Metastatic	Dead	308	male	NA	45	NA	NA
TCGA-ER-A19S-06	Metastatic	Alive	1505	female	NA	81	NA	NA
TCGA-D9-A149-06	Metastatic	Alive	1663	female	NA	65	NA	NO
TCGA-EB-A5SG-06	Metastatic	Alive	2076	female	NA	57	NA	NO
TCGA-GN-A262-06	Metastatic	Alive	4255	female	NA	47	3	NA
TCGA-ER-A19G-06	Metastatic	Alive	9188	female	NA	48	1.6	NO
TCGA-LH-A9QB-06	Metastatic	Alive	11217	female	NA	24	NA	NA
TCGA-EE-A29T-06	Metastatic	Alive	11252	female	NA	51	NA	NA
TCGA-YD-A9TB-06	Metastatic	Alive	NA	female	NA	NA	1.5	NO
TCGA-HR-A5NC-01	Primary Tumor	Alive	NA	female	NA	90	8	NA
TCGA-RP-A6K9-06	Metastatic	Alive	NA	female	NA	NA	NA	NA
TCGA-D9-A1X3-06	Metastatic	Alive	551	male	NA	63	12	YES
TCGA-EB-A24C-01	Primary Tumor	Alive	632	male	NA	56	10	YES
TCGA-GN-A4U8-06	Metastatic	Alive	1487	male	NA	51	NA	NA
TCGA-YD-A9TA-06	Metastatic	Alive	1496	male	NA	75	1.5	NA
TCGA-GN-A265-06	Metastatic	Alive	2948	male	NA	53	NA	NA

TCGA-D9-A148-06	Metastatic	Alive	4609	male	NA	40	NA	NO
TCGA-ER-A19Q-06	Metastatic	Dead	1548	female	NA	37	1.2	NO
TCGA-FW-A3TU-06	Metastatic	Dead	1691	female	NA	72	NA	NA
TCGA-ER-A19W-06	Metastatic	Dead	4507	female	NA	48	0.75	NO
TCGA-ER-A19P-06	Metastatic	Dead	4930	female	NA	47	NA	NA
TCGA-ER-A42H-01	Primary Tumor	Dead	426	male	NA	76	NA	NA
TCGA-ER-A19F-06	Metastatic	Dead	802	male	NA	82	6.7	NA
TCGA-ER-A195-06	Metastatic	Dead	1078	male	NA	46	NA	NA
TCGA-YG-AA3O-06	Metastatic	Dead	1154	male	NA	62	NA	NA
TCGA-ER-A19N-06	Metastatic	Dead	1341	male	NA	47	NA	NA
TCGA-ER-A194-01	Primary Tumor	Dead	1354	male	NA	77	4.5	YES
TCGA-ER-A198-06	Metastatic	Dead	1544	male	NA	45	10	YES
TCGA-ER-A19B-06	Metastatic	Dead	2993	male	NA	42	NA	NA
TCGA-GN-A264-06	Metastatic	Dead	3587	male	NA	60	7	NO
TCGA-ER-A19L-06	Metastatic	Dead	4000	male	NA	35	NA	NA
TCGA-ER-A19H-06	Metastatic	Dead	4634	male	NA	40	0.4	NA
TCGA-ER-A3ES-06	Metastatic	Dead	7514	male	NA	25	NA	NA
TCGA-OD-A75X-06	Metastatic	Dead	9061	male	NA	49	NA	NA
TCGA-D9-A1JW-06	Metastatic	NA	111	male	NA	82	NA	NO
TCGA-EE-A29W-06	Metastatic	Alive	5932	male	stage 0	42	NA	NA
TCGA-D3-A51G-06	Metastatic	Alive	NA	male	stage 0	NA	NA	NA
TCGA-D3-A8GR-06	Metastatic	Dead	3943	female	stage 0	54	0.01	NA
TCGA-D3-A2JB-06	Metastatic	Dead	5110	female	stage 0	70	NA	NA
TCGA-ER-A2NE-06	Metastatic	Dead	613	male	stage 0	39	NA	NA
TCGA-EE-A183-06	Metastatic	Dead	818	male	stage 0	48	NA	NA
TCGA-EE-A20C-06	Metastatic	Dead	4601	male	stage 0	59	NA	NA
TCGA-EE-A2GK-06	Metastatic	Alive	1665	female	stage i	46	0.8	NA
TCGA-D3-A5GN-06	Metastatic	Alive	4129	female	stage i	15	0.6	NA
TCGA-FR-A729-06	Metastatic	Alive	6716	female	stage i	38	0.25	NO
TCGA-EE-A2MP-06	Metastatic	Alive	7563	female	stage i	34	1.05	NA
TCGA-XV-A9W2-01	Primary Tumor	Alive	417	male	stage i	81	1	NA
TCGA-EE-A3J7-06	Metastatic	Alive	1949	male	stage i	43	1.12	YES
TCGA-EE-A20F-06	Metastatic	Alive	2785	male	stage i	53	0.5	NA
TCGA-EE-A2M6-06	Metastatic	Alive	3932	male	stage i	61	0.7	NO
TCGA-EE-A2MR-06	Metastatic	Alive	4088	male	stage i	61	1.25	NO

TCGA-FS-A1ZS-06	Metastatic	Alive	4526	male	stage i	54	1.15	NA
TCGA-EE-A2GE-06	Metastatic	Alive	5286	male	stage i	44	0.9	NO
TCGA-EE-A2GH-06	Metastatic	Alive	6699	male	stage i	34	0.8	NA
TCGA-FS-A1ZJ-06	Metastatic	Dead	1441	female	stage i	75	1.22	NO
TCGA-FS-A1YX-06	Metastatic	Dead	1478	female	stage i	39	1.1	NO
TCGA-EE-A2MM-06	Metastatic	Dead	5107	female	stage i	63	0.6	NA
TCGA-EE-A2MF-06	Metastatic	Dead	8174	female	stage i	39	1.5	NA
TCGA-EE-A2M5-06	Metastatic	Dead	659	male	stage i	49	0.8	NO
TCGA-FS-A1Z4-06	Metastatic	Dead	854	male	stage i	62	0.85	NO
TCGA-EE-A2MN-06	Metastatic	Dead	1446	male	stage i	58	1	NO
TCGA-ER-A19C-06	Metastatic	Dead	1487	male	stage i	77	0.75	NO
TCGA-EE-A2MC-06	Metastatic	Dead	1871	male	stage i	73	1	NO
TCGA-EE-A2MG-06	Metastatic	Dead	3139	male	stage i	23	0.7	NA
TCGA-EE-A2ME-06	Metastatic	Dead	3141	male	stage i	51	0.52	NO
TCGA-W3-AA21-06	Metastatic	Dead	3195	male	stage i	26	1.1	NO
TCGA-D3-A8GS-06	Metastatic	Dead	3564	male	stage i	52	0.6	NA
TCGA-EE-A3JI-06	Metastatic	Dead	4648	male	stage i	48	1.2	NO
TCGA-EE-A20H-06	Metastatic	Dead	5118	male	stage i	56	1.05	NA
TCGA-FS-A4F8-06	Metastatic	Dead	5318	male	stage i	52	0.9	NA
TCGA-DA-A3F5-06	Metastatic	Dead	6873	male	stage i	45	0.4	NA
TCGA-YD-A89C-06	Metastatic	Alive	210	female	stage ia	43	1	NO
TCGA-Z2-AA3V-06	Metastatic	Alive	486	female	stage ia	57	0.5	NO
TCGA-EE-A3AE-06	Metastatic	Alive	1658	female	stage ia	52	0.9	NO
TCGA-EE-A29H-06	Metastatic	Alive	1966	female	stage ia	59	1	NO
TCGA-EE-A2GU-06	Metastatic	Alive	2884	female	stage ia	65	0.3	NO
TCGA-WE-AAA0-06	Metastatic	Alive	1229	male	stage ia	47	0.8	NO
TCGA-EE-A2GI-06	Metastatic	Alive	1482	male	stage ia	39	0.65	NO
TCGA-EE-A2MU-06	Metastatic	Alive	1620	male	stage ia	71	0.5	NO
TCGA-D3-A2JF-06	Metastatic	Alive	1888	male	stage ia	74	0.28	NO
TCGA-EE-A2A6-06	Metastatic	Alive	2620	male	stage ia	43	0.55	NO
TCGA-Z2-AA3S-06	Metastatic	Alive	2950	male	stage ia	58	0.5	NO
TCGA-FR-A8YE-06	Metastatic	Alive	3176	male	stage ia	41	0.79	NO
TCGA-GN-A9SD-06	Metastatic	Dead	1807	female	stage ia	59	0.5	NO
TCGA-FS-A1Z0-06	Metastatic	Dead	6164	female	stage ia	32	0.95	NO
TCGA-3N-A9WB-06	Metastatic	Dead	518	male	stage ia	71	0.7	NO

TCGA-EE-A17X-06	Metastatic	Dead	907	male	stage ia	54	0.8	NO
TCGA-D3-A8GI-06	Metastatic	Dead	1780	male	stage ia	68	0.98	NO
TCGA-EE-A2GJ-06	Metastatic	Dead	2270	male	stage ia	83	0.4	NO
TCGA-GN-A4U5-01	Primary Tumor	Alive	1156	female	stage ib	61	1.15	NO
TCGA-FR-A7UA-06	Metastatic	Alive	1164	female	stage ib	65	1.02	NO
TCGA-EE-A29M-06	Metastatic	Alive	1729	female	stage ib	33	1.02	NO
TCGA-D3-A1Q7-06	Metastatic	Alive	4053	female	stage ib	42	0.68	NO
TCGA-D3-A2JH-06	Metastatic	Alive	1280	male	stage ib	68	1	NO
TCGA-EE-A2MT-06	Metastatic	Alive	2166	male	stage ib	45	1.5	NO
TCGA-DA-A95X-06	Metastatic	Alive	2249	male	stage ib	62	1.45	NO
TCGA-D3-A1QA-06	Metastatic	Alive	2765	male	stage ib	55	1.12	NO
TCGA-D3-A1QA-07	Metastatic	Alive	2765	male	stage ib	55	1.12	NO
TCGA-FR-A3YN-06	Metastatic	Alive	2828	male	stage ib	44	1.25	NO
TCGA-EE-A2A1-06	Metastatic	Alive	3527	male	stage ib	46	1.3	NO
TCGA-D3-A5GU-06	Metastatic	Alive	3808	male	stage ib	36	0.48	NO
TCGA-D3-A5GL-06	Metastatic	Alive	3826	male	stage ib	74	1.6	NO
TCGA-EE-A3JH-06	Metastatic	Alive	4086	male	stage ib	54	1.3	NA
TCGA-ER-A19D-06	Metastatic	Dead	383	female	stage ib	46	1.75	NO
TCGA-ER-A19E-06	Metastatic	Dead	396	female	stage ib	36	1.1	NO
TCGA-EE-A29X-06	Metastatic	Dead	545	female	stage ib	58	2	NO
TCGA-FS-A4F5-06	Metastatic	Dead	874	female	stage ib	77	1.48	NO
TCGA-D3-A3C6-06	Metastatic	Dead	1766	female	stage ib	54	1.3	NO
TCGA-EE-A2GS-06	Metastatic	Dead	2470	female	stage ib	28	1.8	NO
TCGA-EE-A2A5-06	Metastatic	Dead	1195	male	stage ib	43	1.1	NO
TCGA-ER-A2NC-06	Metastatic	Dead	1333	male	stage ib	50	1.97	NO
TCGA-GN-A8LK-06	Metastatic	Dead	1524	male	stage ib	70	0.7	NO
TCGA-EE-A3JA-06	Metastatic	Dead	1618	male	stage ib	44	1.5	NO
TCGA-ER-A19M-06	Metastatic	Dead	1857	male	stage ib	36	1.9	NO
TCGA-D3-A2J8-06	Metastatic	Dead	1992	male	stage ib	48	1.4	NO
TCGA-EE-A184-06	Metastatic	Dead	2073	male	stage ib	72	1.95	NO
TCGA-EE-A29C-06	Metastatic	Dead	2402	male	stage ib	20	1.7	NO
TCGA-EE-A3J3-06	Metastatic	Dead	5237	male	stage ib	42	0.98	NA
TCGA-FS-A1YW-06	Metastatic	Dead	6598	male	stage ib	52	1	NO
TCGA-XV-AB01-06	Metastatic	Alive	403	female	stage ii	54	NA	NA
TCGA-XV-AAZV-01	Primary Tumor	Alive	412	female	stage ii	56	10	NA

TCGA-EE-A2GO-06	Metastatic	Alive	3857	female	stage ii	66	3.4	NO
TCGA-EE-A20B-06	Metastatic	Alive	4070	female	stage ii	66	2.6	NA
TCGA-FR-A44A-06	Metastatic	Alive	5299	female	stage ii	29	2.51	NO
TCGA-XV-A9VZ-01	Primary Tumor	Alive	NA	female	stage ii	90	5	NA
TCGA-EB-A3XB-01	Primary Tumor	Alive	796	male	stage ii	63	NA	NA
TCGA-D3-A5GO-06	Metastatic	Alive	4195	male	stage ii	61	4.3	NA
TCGA-ER-A42L-06	Metastatic	Alive	4533	male	stage ii	49	1.46	NO
TCGA-EE-A2MS-06	Metastatic	Alive	4942	male	stage ii	72	1.8	NO
TCGA-W3-AA1W-06	Metastatic	Alive	6666	male	stage ii	64	1.54	NO
TCGA-D3-A8GJ-06	Metastatic	Alive	7342	male	stage ii	18	2.4	NA
TCGA-XV-AAZW-01	Primary Tumor	Dead	393	female	stage ii	62	13	NA
TCGA-EE-A181-06	Metastatic	Dead	1026	female	stage ii	82	2.5	NA
TCGA-W3-A825-06	Metastatic	Dead	1917	female	stage ii	60	1.3	NA
TCGA-FS-A1ZR-06	Metastatic	Dead	347	male	stage ii	36	2	NA
TCGA-FS-A1ZK-06	Metastatic	Dead	728	male	stage ii	68	5	NA
TCGA-EE-A2M7-06	Metastatic	Dead	877	male	stage ii	66	2.2	NA
TCGA-D3-A8GQ-06	Metastatic	Dead	884	male	stage ii	66	2.6	NA
TCGA-W3-AA1V-06	Metastatic	Dead	1280	male	stage ii	63	1.3	NO
TCGA-EE-A2GR-06	Metastatic	Dead	1301	male	stage ii	78	6.9	NA
TCGA-EE-A2MD-06	Metastatic	Dead	1438	male	stage ii	52	3	YES
TCGA-FS-A1ZB-06	Metastatic	Dead	1486	male	stage ii	57	3	NO
TCGA-FS-A4F4-06	Metastatic	Dead	2028	male	stage ii	64	2	YES
TCGA-FS-A1ZP-06	Metastatic	Dead	2273	male	stage ii	52	2.5	NA
TCGA-W3-AA1R-06	Metastatic	Dead	3379	male	stage ii	71	NA	NA
TCGA-W3-A828-06	Metastatic	Dead	3683	male	stage ii	66	0.5	NA
TCGA-EE-A3J4-06	Metastatic	Dead	3869	male	stage ii	72	2	YES
TCGA-EE-A3AH-06	Metastatic	Dead	4222	male	stage ii	30	3.7	NO
TCGA-EE-A2ML-06	Metastatic	Dead	6590	male	stage ii	35	3	NO
TCGA-EB-A3XE-01	Primary Tumor	Alive	180	female	stage iia	77	3	NO
TCGA-EE-A2GL-06	Metastatic	Alive	2423	female	stage iia	40	3.1	NO
TCGA-EB-A299-01	Primary Tumor	Alive	378	male	stage iia	63	2	YES
TCGA-BF-A3DM-01	Primary Tumor	Alive	601	male	stage iia	63	1.5	YES
TCGA-GN-A4U4-06	Metastatic	Alive	1197	male	stage iia	73	1.7	YES
TCGA-EE-A2GT-06	Metastatic	Alive	1365	male	stage iia	77	2.2	NO
TCGA-WE-A8ZQ-06	Metastatic	Alive	1923	male	stage iia	48	2.3	NO

TCGA-D3-A51R-06	Metastatic	Alive	1941	male	stage iia	60	2.5	NO
TCGA-3N-A9WC-06	Metastatic	Alive	2022	male	stage iia	82	1.8	YES
TCGA-D3-A8GK-06	Metastatic	Alive	5177	male	stage iia	45	2.6	NO
TCGA-EB-A6QZ-01	Primary Tumor	Dead	352	female	stage iia	76	3	NO
TCGA-EE-A2A0-06	Metastatic	Dead	1424	female	stage iia	77	2.2	NO
TCGA-FS-A4FC-06	Metastatic	Dead	1655	female	stage iia	75	4	NO
TCGA-FS-A1YY-06	Metastatic	Dead	6953	female	stage iia	55	2.7	NO
TCGA-WE-A8ZY-06	Metastatic	Dead	1506	male	stage iia	62	3	NO
TCGA-FS-A1ZD-06	Metastatic	Dead	1628	male	stage iia	63	1.18	YES
TCGA-EE-A29S-06	Metastatic	Dead	1864	male	stage iia	79	2.5	NO
TCGA-EE-A2GN-06	Metastatic	Dead	3106	male	stage iia	67	1.9	YES
TCGA-GF-A6C8-06	Metastatic	Alive	62	female	stage iib	62	4	YES
TCGA-EB-A85J-01	Primary Tumor	Alive	360	female	stage iib	66	5.5	NO
TCGA-YG-AA3P-06	Metastatic	Alive	439	female	stage iib	63	10	NO
TCGA-Z2-A8RT-06	Metastatic	Alive	839	female	stage iib	42	4	YES
TCGA-BF-A1PZ-01	Primary Tumor	Alive	853	female	stage iib	71	8	NO
TCGA-FS-A4F0-06	Metastatic	Alive	2367	female	stage iib	67	14	NO
TCGA-EB-A44O-01	Primary Tumor	Alive	81	male	stage iib	69	5	NO
TCGA-D9-A4Z5-01	Primary Tumor	Alive	218	male	stage iib	68	NA	NO
TCGA-BF-AAP2-01	Primary Tumor	Alive	405	male	stage iib	62	3	YES
TCGA-WE-A8K4-01	Primary Tumor	Alive	614	male	stage iib	85	12	NA
TCGA-EB-A4IS-01	Primary Tumor	Alive	774	male	stage iib	77	2.5	YES
TCGA-DA-A960-01	Primary Tumor	Alive	804	male	stage iib	73	2.32	YES
TCGA-WE-A8ZN-06	Metastatic	Alive	1794	male	stage iib	57	4.5	NO
TCGA-EE-A2GC-06	Metastatic	Alive	2051	male	stage iib	82	2.3	YES
TCGA-D3-A3BZ-06	Metastatic	Alive	3976	male	stage iib	63	4.6	NO
TCGA-FS-A1ZZ-06	Metastatic	Dead	822	female	stage iib	54	3.8	YES
TCGA-GN-A268-06	Metastatic	Dead	1910	female	stage iib	83	9.8	NO
TCGA-EE-A29Q-06	Metastatic	Dead	2030	female	stage iib	70	2.3	YES
TCGA-EE-A2GD-06	Metastatic	Dead	10346	female	stage iib	58	4.4	YES
TCGA-EE-A17Z-06	Metastatic	Dead	263	male	stage iib	57	29	NO
TCGA-EB-A5SE-01	Primary Tumor	Dead	401	male	stage iib	73	4	YES
TCGA-FS-A1ZY-06	Metastatic	Dead	824	male	stage iib	71	2.9	YES
TCGA-ER-A193-06	Metastatic	Dead	955	male	stage iib	62	NA	NO
TCGA-FR-A8YC-06	Metastatic	Dead	1059	male	stage iib	78	2.6	YES

TCGA-D3-A2J6-06	Metastatic	Dead	1321	male	stage iib	65	3.2	YES
TCGA-EE-A29B-06	Metastatic	Dead	2588	male	stage iib	67	4	YES
TCGA-D3-A8GM-06	Metastatic	Dead	3259	male	stage iib	73	3.8	YES
TCGA-EE-A2MI-06	Metastatic	Dead	6225	male	stage iib	43	0.4	NA
TCGA-BF-A1PV-01	Primary Tumor	Alive	14	female	stage iic	74	9	YES
TCGA-EB-A82C-01	Primary Tumor	Alive	17	female	stage iic	70	10	YES
TCGA-EB-A3Y6-01	Primary Tumor	Alive	126	female	stage iic	56	4.5	YES
TCGA-EB-A41B-01	Primary Tumor	Alive	291	female	stage iic	76	28	YES
TCGA-BF-AAP7-01	Primary Tumor	Alive	318	female	stage iic	76	5	YES
TCGA-BF-A1PU-01	Primary Tumor	Alive	387	female	stage iic	46	13	YES
TCGA-BF-AAOU-01	Primary Tumor	Alive	476	female	stage iic	73	12	YES
TCGA-BF-A5ES-01	Primary Tumor	Alive	490	female	stage iic	76	8	YES
TCGA-EB-A44P-01	Primary Tumor	Alive	741	female	stage iic	58	NA	NO
TCGA-EB-A4XL-01	Primary Tumor	Alive	777	female	stage iic	56	7	YES
TCGA-EB-A5UM-01	Primary Tumor	Alive	779	female	stage iic	48	5	YES
TCGA-EB-A3XD-01	Primary Tumor	Alive	1160	female	stage iic	53	NA	NA
TCGA-EE-A29P-06	Metastatic	Alive	1716	female	stage iic	73	4.5	YES
TCGA-ER-A196-01	Primary Tumor	Alive	1785	female	stage iic	64	22	YES
TCGA-DA-A95V-06	Metastatic	Alive	2193	female	stage iic	83	4.1	YES
TCGA-EE-A2GM-06	Metastatic	Alive	2296	female	stage iic	70	6.5	YES
TCGA-D3-A3CC-06	Metastatic	Alive	2644	female	stage iic	69	7.2	YES
TCGA-GF-A2C7-01	Primary Tumor	Alive	21	male	stage iic	48	NA	YES
TCGA-EB-A3HV-01	Primary Tumor	Alive	39	male	stage iic	37	13	YES
TCGA-EB-A553-01	Primary Tumor	Alive	226	male	stage iic	62	10	YES
TCGA-EB-A3XF-01	Primary Tumor	Alive	278	male	stage iic	57	10	YES
TCGA-YG-AA3N-01	Primary Tumor	Alive	306	male	stage iic	67	17	NA
TCGA-BF-A5EQ-01	Primary Tumor	Alive	323	male	stage iic	63	5	NA
TCGA-BF-A5ER-01	Primary Tumor	Alive	327	male	stage iic	63	14	YES
TCGA-BF-AAP4-01	Primary Tumor	Alive	335	male	stage iic	61	15	YES
TCGA-EB-A85I-01	Primary Tumor	Alive	362	male	stage iic	66	10	YES
TCGA-EB-A6QY-01	Primary Tumor	Alive	382	male	stage iic	71	10	YES
TCGA-BF-AAP1-01	Primary Tumor	Alive	409	male	stage iic	86	15	YES
TCGA-EB-A97M-01	Primary Tumor	Alive	414	male	stage iic	66	15	YES
TCGA-BF-A9VF-01	Primary Tumor	Alive	440	male	stage iic	77	12	YES
TCGA-BF-AAOX-01	Primary Tumor	Alive	444	male	stage iic	83	11	YES

TCGA-BF-AAP8-01	Primary Tumor	Alive	447	male	stage iic	58	6	YES
TCGA-IH-A3EA-01	Primary Tumor	Alive	524	male	stage iic	61	NA	YES
TCGA-EB-A431-01	Primary Tumor	Alive	568	male	stage iic	34	15	YES
TCGA-EB-A3XC-01	Primary Tumor	Alive	650	male	stage iic	74	NA	YES
TCGA-FR-A3R1-01	Primary Tumor	Alive	685	male	stage iic	69	6.3	YES
TCGA-BF-A5EO-01	Primary Tumor	Alive	703	male	stage iic	65	8	YES
TCGA-GN-A8LN-01	Primary Tumor	Alive	772	male	stage iic	68	4.85	YES
TCGA-BF-A1Q0-01	Primary Tumor	Alive	831	male	stage iic	80	5	YES
TCGA-EB-A51B-01	Primary Tumor	Alive	931	male	stage iic	53	10	YES
TCGA-EB-A1NK-01	Primary Tumor	Alive	1039	male	stage iic	48	8	YES
TCGA-EB-A5UN-06	Metastatic	Alive	1792	male	stage iic	49	14	YES
TCGA-EB-A430-01	Primary Tumor	Alive	NA	male	stage iic	83	18	YES
TCGA-EB-A41A-01	Primary Tumor	Alive	NA	male	stage iic	90	7	YES
TCGA-EB-A550-01	Primary Tumor	Dead	264	female	stage iic	75	15	YES
TCGA-FR-A2OS-01	Primary Tumor	Dead	368	female	stage iic	49	NA	YES
TCGA-EB-A5SF-01	Primary Tumor	Dead	369	female	stage iic	78	11	YES
TCGA-ER-A19K-01	Primary Tumor	Dead	469	female	stage iic	79	6.8	YES
TCGA-FS-A1ZF-06	Metastatic	Dead	470	female	stage iic	78	4.3	YES
TCGA-EB-A6R0-01	Primary Tumor	Dead	608	female	stage iic	58	10	YES
TCGA-GN-A8LL-06	Metastatic	Dead	650	female	stage iic	68	5	YES
TCGA-EB-A42Y-01	Primary Tumor	Dead	721	female	stage iic	73	5	YES
TCGA-FS-A1ZU-06	Metastatic	Dead	808	female	stage iic	70	8.4	YES
TCGA-FR-A8YD-06	Metastatic	Dead	1103	female	stage iic	56	4.5	YES
TCGA-GN-A26D-06	Metastatic	Dead	1460	female	stage iic	72	12	YES
TCGA-FS-A4F2-06	Metastatic	Dead	1525	female	stage iic	46	4.7	YES
TCGA-DA-A1I8-06	Metastatic	Dead	1640	female	stage iic	63	8	YES
TCGA-FR-A726-01	Primary Tumor	Dead	0	male	stage iic	90	14	YES
TCGA-EB-A44N-01	Primary Tumor	Dead	205	male	stage iic	59	5	YES
TCGA-EB-A4P0-01	Primary Tumor	Dead	326	male	stage iic	82	8	YES
TCGA-DA-A95Y-06	Metastatic	Dead	430	male	stage iic	68	5.03	YES
TCGA-D3-A1Q3-06	Metastatic	Dead	507	male	stage iic	64	4.6	YES
TCGA-GF-A769-01	Primary Tumor	Dead	1070	male	stage iic	39	74	YES
TCGA-FS-A1ZE-06	Metastatic	Dead	1413	male	stage iic	40	5	YES
TCGA-EB-A82B-01	Primary Tumor	Alive	390	female	stage iii	58	20	YES
TCGA-FW-A5DY-06	Metastatic	Alive	587	female	stage iii	48	NA	NA

TCGA-D3-A3C7-06	Metastatic	Alive	1429	female	stage iii	57	NA	NA
TCGA-EB-A5SH-06	Metastatic	Alive	1643	female	stage iii	60	5	YES
TCGA-D3-A2JC-06	Metastatic	Alive	2639	female	stage iii	53	NA	NA
TCGA-D3-A1QB-06	Metastatic	Alive	2912	female	stage iii	75	NA	NA
TCGA-D3-A5GR-06	Metastatic	Alive	5424	female	stage iii	23	0.98	NA
TCGA-EE-A2MK-06	Metastatic	Alive	5487	female	stage iii	18	5.2	NO
TCGA-EE-A3JB-06	Metastatic	Alive	6138	female	stage iii	60	1.86	NA
TCGA-BF-AAP6-01	Primary Tumor	Alive	325	male	stage iii	55	20	YES
TCGA-EB-A5UL-06	Metastatic	Alive	891	male	stage iii	71	4	NA
TCGA-FW-A3R5-06	Metastatic	Alive	1124	male	stage iii	68	NA	NA
TCGA-FS-A1ZT-06	Metastatic	Alive	1617	male	stage iii	55	1.15	NA
TCGA-EE-A3AC-06	Metastatic	Alive	1948	male	stage iii	47	NA	NA
TCGA-FS-A1ZM-06	Metastatic	Alive	3080	male	stage iii	74	1.2	NA
TCGA-D3-A3MR-06	Metastatic	Alive	3151	male	stage iii	42	NA	NA
TCGA-GN-A4U3-06	Metastatic	Alive	3708	male	stage iii	30	3	NO
TCGA-EE-A3AB-06	Metastatic	Alive	3733	male	stage iii	30	NA	NA
TCGA-EE-A3AA-06	Metastatic	Alive	3781	male	stage iii	47	NA	NA
TCGA-DA-A1HY-06	Metastatic	Alive	4407	male	stage iii	42	1.13	YES
TCGA-D3-A51J-06	Metastatic	Alive	4414	male	stage iii	19	NA	NA
TCGA-D3-A8GP-06	Metastatic	Alive	4638	male	stage iii	77	1.8	NA
TCGA-DA-A1I1-06	Metastatic	Alive	6768	male	stage iii	55	NA	NA
TCGA-EE-A2M8-06	Metastatic	Dead	601	female	stage iii	54	2	NO
TCGA-FS-A4FB-06	Metastatic	Dead	813	female	stage iii	46	2	NA
TCGA-D3-A3CE-06	Metastatic	Dead	1832	female	stage iii	74	NA	NA
TCGA-D3-A2JN-06	Metastatic	Dead	2022	female	stage iii	46	NA	NA
TCGA-W3-AA1O-06	Metastatic	Dead	122	male	stage iii	85	NA	NA
TCGA-D3-A3MO-06	Metastatic	Dead	284	male	stage iii	47	NA	NA
TCGA-EE-A2MH-06	Metastatic	Dead	516	male	stage iii	66	3.4	NO
TCGA-EB-A5KH-06	Metastatic	Dead	619	male	stage iii	55	NA	NA
TCGA-EE-A3JD-06	Metastatic	Dead	832	male	stage iii	70	NA	NA
TCGA-EE-A3AD-06	Metastatic	Dead	875	male	stage iii	50	NA	NA
TCGA-EE-A3J5-06	Metastatic	Dead	1124	male	stage iii	71	4.8	NO
TCGA-EE-A3AG-06	Metastatic	Dead	1265	male	stage iii	25	NA	NA
TCGA-ER-A3EV-06	Metastatic	Dead	1429	male	stage iii	55	4.4	NA
TCGA-W3-AA1Q-06	Metastatic	Dead	2101	male	stage iii	57	NA	NA

TCGA-D3-A1Q6-06	Metastatic	Dead	2184	male	stage iii	55	50	NA
TCGA-EE-A180-06	Metastatic	Dead	2889	male	stage iii	69	4.9	NO
TCGA-EE-A2MJ-06	Metastatic	Dead	2927	male	stage iii	60	11	NO
TCGA-DA-A1I2-06	Metastatic	Dead	5370	male	stage iii	45	NA	YES
TCGA-D9-A6E9-06	Metastatic	Alive	301	female	stage iiia	75	4	NO
TCGA-D3-A3MU-06	Metastatic	Alive	1209	male	stage iiia	53	2.9	NO
TCGA-D9-A6EC-06	Metastatic	Alive	2359	male	stage iiia	56	3	NO
TCGA-D3-A2JA-06	Metastatic	Alive	3514	male	stage iiia	68	1.2	NO
TCGA-GN-A26A-06	Metastatic	Dead	988	female	stage iiia	63	2.3	NO
TCGA-EE-A2MQ-06	Metastatic	Dead	1315	female	stage iiia	70	3.1	NO
TCGA-ER-A3ET-06	Metastatic	Dead	2829	female	stage iiia	64	3.55	NA
TCGA-D3-A2JG-06	Metastatic	Dead	3453	female	stage iiia	30	2.5	NO
TCGA-3N-A9WD-06	Metastatic	Dead	395	male	stage iiia	82	1.25	NO
TCGA-D3-A3ML-06	Metastatic	Dead	422	male	stage iiia	70	2.3	NO
TCGA-D9-A6EG-06	Metastatic	Dead	698	male	stage iiia	56	5	YES
TCGA-FS-A1ZN-01	Primary Tumor	Dead	730	male	stage iiia	43	8	YES
TCGA-EE-A3J8-06	Metastatic	Dead	1044	male	stage iiia	59	4.8	NO
TCGA-EE-A29A-06	Metastatic	Dead	1927	male	stage iiia	68	2.3	NO
TCGA-GN-A267-06	Metastatic	Dead	1960	male	stage iiia	38	5.2	NO
TCGA-EE-A29G-06	Metastatic	Dead	2192	male	stage iiia	53	7.4	NO
TCGA-EB-A5VV-06	Metastatic	Alive	214	female	stage iiib	74	4	YES
TCGA-FW-A3TV-06	Metastatic	Alive	411	female	stage iiib	57	0.5	NA
TCGA-BF-A3DJ-01	Primary Tumor	Alive	464	female	stage iiib	36	11	YES
TCGA-FR-A728-01	Primary Tumor	Alive	583	female	stage iiib	54	12	YES
TCGA-D9-A3Z3-06	Metastatic	Alive	678	female	stage iiib	39	3.9	NO
TCGA-BF-A3DL-01	Primary Tumor	Alive	769	female	stage iiib	84	3	YES
TCGA-EB-A4OY-01	Primary Tumor	Alive	977	female	stage iiib	65	10	NA
TCGA-D3-A3MV-06	Metastatic	Alive	1378	female	stage iiib	38	1.2	YES
TCGA-WE-A8ZO-06	Metastatic	Alive	2145	female	stage iiib	73	2.4	NO
TCGA-DA-A1HV-06	Metastatic	Alive	2329	female	stage iiib	75	NA	NA
TCGA-GF-A6C9-06	Metastatic	Alive	480	male	stage iiib	78	NA	NA
TCGA-WE-A8K6-06	Metastatic	Alive	546	male	stage iiib	79	NA	NA
TCGA-EB-A24D-01	Primary Tumor	Alive	645	male	stage iiib	72	36	NO
TCGA-WE-A8JZ-06	Metastatic	Alive	731	male	stage iiib	70	14	YES
TCGA-D3-A51K-06	Metastatic	Alive	1002	male	stage iiib	51	NA	NA

TCGA-WE-A8ZX-06	Metastatic	Alive	1089	male	stage iiib	45	NA	NA
TCGA-DA-A95W-06	Metastatic	Alive	1136	male	stage iiib	52	NA	NA
TCGA-DA-A3F8-06	Metastatic	Alive	1319	male	stage iiib	39	1.9	NO
TCGA-FS-A1ZW-06	Metastatic	Alive	1505	male	stage iiib	65	1.2	YES
TCGA-EE-A3JE-06	Metastatic	Alive	1562	male	stage iiib	75	3.7	YES
TCGA-EE-A2GB-06	Metastatic	Alive	1803	male	stage iiib	51	1.65	YES
TCGA-EE-A29E-06	Metastatic	Alive	1940	male	stage iiib	54	3.2	NO
TCGA-DA-A1I7-06	Metastatic	Alive	2703	male	stage iiib	62	NA	NA
TCGA-WE-A8ZM-06	Metastatic	Alive	3082	male	stage iiib	70	NA	NA
TCGA-EB-A3Y7-01	Primary Tumor	Dead	326	female	stage iiib	86	4	NO
TCGA-ER-A197-06	Metastatic	Dead	424	female	stage iiib	83	8	YES
TCGA-EB-A4IQ-01	Primary Tumor	Dead	636	female	stage iiib	42	15	YES
TCGA-FS-A1ZA-06	Metastatic	Dead	843	female	stage iiib	45	4.5	YES
TCGA-DA-A1HW-06	Metastatic	Dead	1096	female	stage iiib	37	0.7	NO
TCGA-HR-A2OH-06	Metastatic	Dead	2004	female	stage iiib	46	3.4	YES
TCGA-DA-A1IA-06	Metastatic	Dead	2005	female	stage iiib	32	1.3	NO
TCGA-BF-A1PX-01	Primary Tumor	Dead	282	male	stage iiib	56	12	YES
TCGA-EB-A44R-06	Metastatic	Dead	315	male	stage iiib	52	NA	NA
TCGA-DA-A3F3-06	Metastatic	Dead	319	male	stage iiib	52	NA	NA
TCGA-EB-A5VU-01	Primary Tumor	Dead	321	male	stage iiib	56	15	YES
TCGA-EE-A2GP-06	Metastatic	Dead	423	male	stage iiib	80	4.2	YES
TCGA-EB-A57M-01	Primary Tumor	Dead	472	male	stage iiib	56	8	YES
TCGA-EE-A17Y-06	Metastatic	Dead	828	male	stage iiib	69	2.8	YES
TCGA-ER-A2NB-01	Primary Tumor	Dead	857	male	stage iiib	57	4.39	YES
TCGA-ER-A2NF-01	Primary Tumor	Dead	877	male	stage iiib	53	4	YES
TCGA-ER-A2NF-06	Metastatic	Dead	877	male	stage iiib	53	4	YES
TCGA-D3-A8GB-06	Metastatic	Dead	938	male	stage iiib	48	3.5	NO
TCGA-D3-A1Q9-06	Metastatic	Dead	961	male	stage iiib	72	6	YES
TCGA-DA-A3F2-06	Metastatic	Dead	1032	male	stage iiib	55	5.1	NO
TCGA-DA-A1IC-06	Metastatic	Dead	2071	male	stage iiib	81	2.5	NO
TCGA-D3-A8GL-06	Metastatic	Dead	2711	male	stage iiib	43	1.32	NO
TCGA-ER-A19O-06	Metastatic	Dead	NA	male	stage iiib	56	NA	NA
TCGA-GF-A3OT-06	Metastatic	Alive	301	female	stage iiic	58	NA	NA
TCGA-BF-A5EP-01	Primary Tumor	Alive	335	female	stage iiic	75	70	YES
TCGA-XV-AAZY-01	Primary Tumor	Alive	405	female	stage iiic	76	16	NA

TCGA-EB-A44Q-06	Metastatic	Alive	422	female	stage iiic	51	NA	YES
TCGA-EE-A29R-06	Metastatic	Alive	440	female	stage iiic	48	3.5	YES
TCGA-FR-A69P-06	Metastatic	Alive	478	female	stage iiic	34	NA	NA
TCGA-D9-A4Z3-01	Primary Tumor	Alive	505	female	stage iiic	73	75	YES
TCGA-FR-A7U9-06	Metastatic	Alive	571	female	stage iiic	63	2.8	YES
TCGA-EB-A551-01	Primary Tumor	Alive	590	female	stage iiic	78	10	YES
TCGA-GF-A4EO-06	Metastatic	Alive	591	female	stage iiic	74	NA	NA
TCGA-EB-A4OZ-01	Primary Tumor	Alive	620	female	stage iiic	41	18	NO
TCGA-WE-AAA3-06	Metastatic	Alive	651	female	stage iiic	84	7	YES
TCGA-BF-A3DN-01	Primary Tumor	Alive	717	female	stage iiic	81	3	YES
TCGA-D3-A8GD-06	Metastatic	Alive	718	female	stage iiic	63	20	YES
TCGA-WE-AAA4-06	Metastatic	Alive	760	female	stage iiic	56	NA	NA
TCGA-D3-A51T-06	Metastatic	Alive	818	female	stage iiic	59	4.2	YES
TCGA-D3-A3C8-06	Metastatic	Alive	1409	female	stage iiic	58	NA	NA
TCGA-D3-A2JO-06	Metastatic	Alive	2010	female	stage iiic	50	NA	YES
TCGA-D3-A1Q4-06	Metastatic	Alive	3408	female	stage iiic	53	1.45	YES
TCGA-QB-A6FS-06	Metastatic	Alive	220	male	stage iiic	49	NA	NA
TCGA-WE-AA9Y-06	Metastatic	Alive	370	male	stage iiic	37	1.4	NO
TCGA-EB-A42Z-01	Primary Tumor	Alive	441	male	stage iiic	49	10	YES
TCGA-D3-A5GT-01	Primary Tumor	Alive	487	male	stage iiic	43	1.92	YES
TCGA-FW-A5DX-01	Primary Tumor	Alive	640	male	stage iiic	71	7	NO
TCGA-D9-A6EA-06	Metastatic	Alive	766	male	stage iiic	70	6	NO
TCGA-FR-A7U8-06	Metastatic	Alive	847	male	stage iiic	50	NA	NA
TCGA-FS-A4F9-06	Metastatic	Alive	1035	male	stage iiic	80	10	YES
TCGA-EB-A6L9-06	Metastatic	Alive	1109	male	stage iiic	55	NA	YES
TCGA-ER-A2NH-06	Metastatic	Alive	1264	male	stage iiic	49	4	NO
TCGA-WE-A8K1-06	Metastatic	Alive	1492	male	stage iiic	74	3	YES
TCGA-D3-A51F-06	Metastatic	Alive	1695	male	stage iiic	51	7	YES
TCGA-D3-A2JP-06	Metastatic	Alive	1812	male	stage iiic	37	NA	NA
TCGA-EE-A2A2-06	Metastatic	Alive	1814	male	stage iiic	71	7	YES
TCGA-ER-A1A1-06	Metastatic	Alive	3196	male	stage iiic	58	NA	NA
TCGA-EE-A185-06	Metastatic	Dead	151	female	stage iiic	55	6	YES
TCGA-ER-A199-06	Metastatic	Dead	279	female	stage iiic	86	3	YES
TCGA-FS-A1ZG-06	Metastatic	Dead	295	female	stage iiic	60	6	YES
TCGA-GN-A4U7-06	Metastatic	Dead	317	female	stage iiic	56	1.39	NO

TCGA-ER-A42K-06	Metastatic	Dead	394	female	stage iiic	40	5.5	YES
TCGA-EE-A3AF-06	Metastatic	Dead	420	female	stage iiic	48	NA	NA
TCGA-EE-A182-06	Metastatic	Dead	447	female	stage iiic	84	14	YES
TCGA-D3-A1Q1-06	Metastatic	Dead	504	female	stage iiic	79	0.4	NO
TCGA-ER-A2ND-06	Metastatic	Dead	710	female	stage iiic	57	1	NO
TCGA-D3-A3CF-06	Metastatic	Dead	746	female	stage iiic	61	9	YES
TCGA-D3-A2JE-06	Metastatic	Dead	841	female	stage iiic	75	NA	NA
TCGA-DA-A1IB-06	Metastatic	Dead	1235	female	stage iiic	69	1.5	YES
TCGA-ER-A2NG-06	Metastatic	Dead	1490	female	stage iiic	43	3	YES
TCGA-EE-A29L-06	Metastatic	Dead	79	male	stage iiic	78	7	YES
TCGA-FS-A1Z7-06	Metastatic	Dead	237	male	stage iiic	19	17	YES
TCGA-WE-A8ZR-06	Metastatic	Dead	274	male	stage iiic	49	7.3	YES
TCGA-D3-A2JD-06	Metastatic	Dead	361	male	stage iiic	58	18	YES
TCGA-D3-A2JK-06	Metastatic	Dead	368	male	stage iiic	24	4.3	YES
TCGA-EE-A29D-06	Metastatic	Dead	425	male	stage iiic	87	3.2	YES
TCGA-D9-A3Z1-06	Metastatic	Dead	468	male	stage iiic	66	1.7	NA
TCGA-D9-A3Z4-01	Primary Tumor	Dead	519	male	stage iiic	54	12	YES
TCGA-QB-AA9O-06	Metastatic	Dead	549	male	stage iiic	73	NA	NA
TCGA-D9-A4Z6-06	Metastatic	Dead	561	male	stage iiic	54	NA	NA
TCGA-GN-A4U9-06	Metastatic	Dead	673	male	stage iiic	71	1.7	YES
TCGA-D3-A2J9-06	Metastatic	Dead	723	male	stage iiic	75	21	YES
TCGA-EE-A29V-06	Metastatic	Dead	787	male	stage iiic	85	3.5	YES
TCGA-GN-A26C-01	Primary Tumor	Dead	821	male	stage iiic	77	14	YES
TCGA-DA-A1I4-06	Metastatic	Dead	1093	male	stage iiic	51	3.4	YES
TCGA-D3-A8GC-06	Metastatic	Dead	2421	male	stage iiic	48	NA	NA
TCGA-FS-A4FD-06	Metastatic	Dead	2454	male	stage iiic	39	2	NA
TCGA-D3-A2J7-06	Metastatic	Dead	3136	male	stage iiic	67	3.6	YES
TCGA-WE-A8ZT-06	Metastatic	Alive	359	female	stage iv	25	4	YES
TCGA-BF-AAP0-06	Metastatic	Alive	454	female	stage iv	40	NA	NA
TCGA-FW-A3I3-06	Metastatic	Alive	531	female	stage iv	59	NA	NA
TCGA-D3-A51N-06	Metastatic	Alive	688	female	stage iv	56	NA	NA
TCGA-DA-A1I5-06	Metastatic	Alive	4107	female	stage iv	27	0.6	NO
TCGA-RP-A693-06	Metastatic	Alive	10	male	stage iv	77	NA	NA
TCGA-RP-A694-06	Metastatic	Alive	21	male	stage iv	71	NA	NA
TCGA-DA-A95Z-06	Metastatic	Alive	396	male	stage iv	87	NA	NA

TCGA-D3-A5GS-06	Metastatic	Alive	553	male	stage iv	58	0.62	NO
TCGA-D3-A8GE-06	Metastatic	Alive	804	male	stage iv	26	NA	NA
TCGA-ER-A3PL-06	Metastatic	Alive	1010	male	stage iv	30	2.2	YES
TCGA-ER-A19A-06	Metastatic	Alive	2365	male	stage iv	79	NA	NA
TCGA-RP-A695-06	Metastatic	Alive	NA	male	stage iv	NA	NA	NA
TCGA-EB-A5FP-01	Primary Tumor	Dead	454	female	stage iv	65	8	YES
TCGA-FS-A1Z3-06	Metastatic	Dead	636	female	stage iv	72	NA	NA
TCGA-FS-A1ZH-06	Metastatic	Dead	996	female	stage iv	71	3	YES
TCGA-ER-A19J-06	Metastatic	Dead	196	male	stage iv	54	NA	NA
TCGA-ER-A19T-01	Primary Tumor	Dead	270	male	stage iv	51	15	NO
TCGA-ER-A19T-06	Metastatic	Dead	270	male	stage iv	51	15	NO
TCGA-EE-A20I-06	Metastatic	Dead	412	male	stage iv	79	NA	YES
TCGA-GN-A263-01	Primary Tumor	Dead	467	male	stage iv	24	NA	YES
TCGA-DA-A1I0-06	Metastatic	Dead	620	male	stage iv	63	4.5	YES
TCGA-D3-A1Q8-06	Metastatic	Dead	854	male	stage iv	33	NA	NA
TCGA-WE-A8K5-06	Metastatic	Dead	1860	male	stage iv	65	1.7	NO

Table S2 Common differentially expressed genes in all comparisons. The gene expression comparison was made between normal skin and all tumor samples, normal skin and primary tumors, normal skin and metastatic tumors, and primary tumors and metastatic tumors

Gene	GeneCards Summary
AGR3	AGR3 (Anterior Gradient 3, Protein Disulphide Isomerase Family Member) is a Protein Coding gene. Diseases associated with AGR3 include Breast Abscess and Ritter's Disease. Gene Ontology (GO) annotations related to this gene include dystroglycan binding. An important paralog of this gene is AGR2.
CRP	CRP (C-Reactive Protein) is a Protein Coding gene. Diseases associated with CRP include Appendicitis and Acute Pyelonephritis. Among its related pathways are Folate Metabolism and Activation of cAMP-Dependent PKA. Gene Ontology (GO) annotations related to this gene include calcium ion binding and cholesterol binding. An important paralog of this gene is APCS.
HTN3	HTN3 (Histatin 3) is a Protein Coding gene. Diseases associated with HTN3 include Oral Candidiasis and Candidiasis. Among its related pathways are Innate Immune System and Defensins. An important paralog of this gene is HTN1.
KRT26	KRT26 (Keratin 26) is a Protein Coding gene. Among its related pathways are Relaxin signaling pathway and Developmental Biology. Gene Ontology (GO) annotations related to this gene include structural molecule activity. An important paralog of this gene is KRT27.
KRT38	KRT38 (Keratin 38) is a Protein Coding gene. Diseases associated with KRT38 include Nodular Basal Cell Carcinoma and Hair Follicle Neoplasm. Among its related pathways are Relaxin signaling pathway and Developmental Biology. Gene Ontology (GO) annotations related to this gene include structural molecule activity. An important paralog of this gene is KRT37.
KRTAP10-3	KRTAP10-3 (Keratin Associated Protein 10-3) is a Protein Coding gene. Among its related pathways are Developmental Biology and Keratinization. An important paralog of this gene is KRTAP10-11.
KRTAP10-4	KRTAP10-4 (Keratin Associated Protein 10-4) is a Protein Coding gene. Among its related pathways are Developmental Biology and Keratinization. An important paralog of this gene is KRTAP10-11.
KRTAP10-5	KRTAP10-5 (Keratin Associated Protein 10-5) is a Protein Coding gene. Diseases associated with KRTAP10-5 include Three M Syndrome 1. Among its related pathways are Developmental Biology and Keratinization. Gene Ontology (GO) annotations related to this gene include identical protein binding. An important paralog of this gene is KRTAP10-1.
KRTAP10-7	KRTAP10-7 (Keratin Associated Protein 10-7) is a Protein Coding gene. Diseases associated with KRTAP10-7 include Trochlear Nerve Disease and Vulvitis. Among its related pathways are Developmental Biology and Keratinization. Gene Ontology (GO) annotations related to this gene include identical protein binding. An important paralog of this gene is KRTAP10-6.
KRTAP10-8	KRTAP10-8 (Keratin Associated Protein 10-8) is a Protein Coding gene. Diseases associated with KRTAP10-8 include Trochlear Nerve Disease and Vulvitis. Among its related pathways are Developmental Biology and Keratinization. An important paralog of this gene is KRTAP10-1.
KRTAP10-9	KRTAP10-9 (Keratin Associated Protein 10-9) is a Protein Coding gene. Diseases associated with KRTAP10-9 include Three M Syndrome 1. Among its related pathways are Developmental Biology and Keratinization. An important paralog of this gene is KRTAP10-11.
KRTAP1-1	KRTAP1-1 (Keratin Associated Protein 1-1) is a Protein Coding gene. Diseases associated with KRTAP1-1 include Pulmonary Embolism And Infarction and Tuberculous Empyema. Among its related pathways are Developmental Biology and Keratinization. An important paralog of this gene is KRTAP1-3.
KRTAP2-4	KRTAP2-4 (Keratin Associated Protein 2-4) is a Protein Coding gene. Among its related pathways are Developmental Biology and Keratinization. An important paralog of this gene is KRTAP2-3.

KRTAP4-5	KRTAP4-5 (Keratin Associated Protein 4-5) is a Protein Coding gene. Diseases associated with KRTAP4-5 include Hypopharynx Cancer. Among its related pathways are Developmental Biology and Keratinization. An important paralog of this gene is KRTAP4-12.
PRH2	PRH2 (Proline Rich Protein HaeIII Subfamily 2) is a Protein Coding gene. Diseases associated with PRH2 include Dental Caries and Hydrolethalus Syndrome 1. Among its related pathways are Salivary secretion. An important paralog of this gene is PRH1.
SAGE1	SAGE1 (Sarcoma Antigen 1) is a Protein Coding gene. Diseases associated with SAGE1 include Acute Laryngopharyngitis and Sarcoma. An important paralog of this gene is INTS6L.
TBC1D3	TBC1D3 (TBC1 Domain Family Member 3) is a Protein Coding gene. Diseases associated with TBC1D3 include Prostate Cancer. Among its related pathways are TBC/RABGAPs and Vesicle-mediated transport. Gene Ontology (GO) annotations related to this gene include GTPase activator activity. An important paralog of this gene is TBC1D3C.
TKTL1	TKTL1 (Transketolase Like 1) is a Protein Coding gene. Diseases associated with TKTL1 include Wernicke-Korsakoff Syndrome and Conjunctival Nevus. Among its related pathways are Metabolism and Carbon metabolism. Gene Ontology (GO) annotations related to this gene include oxidoreductase activity, acting on the aldehyde or oxo group of donors, disulfide as acceptor and transketolase activity. An important paralog of this gene is TKTL2

Table S3 Eleven genes were significantly differentially expressed in the comparison of metastatic tumors with primary tumors

Gene	GeneCards Summary
HMX1	HMX1 (H6 Family Homeobox 1) is a Protein Coding gene. Diseases associated with HMX1 include Oculoauricular Syndrome and Anterior Segment Dysgenesis. Gene Ontology (GO) annotations related to this gene include sequence-specific DNA binding. An important paralog of this gene is HMX3.
ALB	ALB (Albumin) is a Protein Coding gene. Diseases associated with ALB include Analbuminemia and Hyperthyroxinemia, Familial Dysalbuminemic. Among its related pathways are Lipoprotein metabolism and Folate Metabolism. Gene Ontology (GO) annotations related to this gene include enzyme binding and chaperone binding. An important paralog of this gene is AFP.
ORM2	ORM2 (Orosomucoid 2) is a Protein Coding gene. Among its related pathways are Response to elevated platelet cytosolic Ca2+ and Innate Immune System. An important paralog of this gene is ORM1.
PRB3	PRB3 (Proline Rich Protein BstNI Subfamily 3) is a Protein Coding gene. Diseases associated with PRB3 include Cataract 21, Multiple Types. An important paralog of this gene is PRB2.
SFTA3	SFTA3 (Surfactant Associated 3) is an RNA Gene, and is affiliated with the lncRNA class. Diseases associated with SFTA3 include Spherocytosis, Type 1 and Spherocytosis, Type 2. Among its related pathways are Defective CSF2RA causes pulmonary surfactant metabolism dysfunction 4 (SMDP4) and Surfactant metabolism.
RTL1	RTL1 (Retrotransposon Gag Like 1) is a Protein Coding gene. Diseases associated with RTL1 include Temple Syndrome and Kagami-Ogata Syndrome. An important paralog of this gene is RTL3.
CRABP1	CRABP1 (Cellular Retinoic Acid Binding Protein 1) is a Protein Coding gene. Diseases associated with CRABP1 include Teratocarcinoma and Embryonal Carcinoma. Among its related pathways are Signaling by GPCR and Vitamin A and Carotenoid Metabolism. Gene Ontology (GO) annotations related to this gene include transporter activity and retinol binding. An important paralog of this gene is CRABP2.
OR1E1	OR1E1 (Olfactory Receptor Family 1 Subfamily E Member 1) is a Protein Coding gene. Diseases associated with OR1E1 include Plasminogen Deficiency, Type I and Pseudobulbar Palsy. Among its related pathways are Signaling by GPCR and GPCRs, Other. Gene Ontology (GO) annotations related to this gene include G protein-coupled receptor activity and olfactory receptor activity. An important paralog of this gene is OR1E2.
APOH	APOH (Apolipoprotein H) is a Protein Coding gene. Diseases associated with APOH include Antiphospholipid Syndrome and Syphilis. Among its related pathways are Response to elevated platelet cytosolic Ca2+ and Statin Pathway. Gene Ontology (GO) annotations related to this gene include identical protein binding and heparin binding. An important paralog of this gene is CR2.
FBN3	FBN3 (Fibrillin 3) is a Protein Coding gene. Diseases associated with FBN3 include Weill-Marchesani Syndrome and Marfan Syndrome. Among its related pathways are Integrin Pathway and Degradation of the extracellular matrix. Gene Ontology (GO) annotations related to this gene include calcium ion binding and extracellular matrix structural constituent. An important paralog of this gene is FBN2.
IGFL1	IGFL1 (IGF Like Family Member 1) is a Protein Coding gene. An important paralog of this gene is IGFL3.

Table S4 Prognostic genes in patients with metastatic melanoma identified using the GSE65904 dataset

**Gene**	**HR**	**HR.95L**	**HR.95H**	**P value**
ELL2	0.6616051	0.4629348	0.9455356	0.0233683
RTP4	0.7496163	0.5731019	0.9804968	0.0354012
GPR143	1.1907784	1.0078854	1.4068596	0.0401421
TTYH2	1.467523	1.0105642	2.1311104	0.0438865

Abbreviations: HR hazard ratio; Analysis of a Cox proportional hazards model was performed after adjusting for sex and age upon diagnosis

Table S5 Prognostic genes in patients with metastatic melanoma identified using the GSE22153 dataset

Gene	HR	HR.95L	HR.95H	P value
S100A1	0.8901963	0.8092184	0.9792776	0.0168349
RTP4	0.7619647	0.5996046	0.9682885	0.0261796
SLC45A2	0.903176	0.8214021	0.9930908	0.0354528
THRB	2.0757028	1.0071414	4.2779909	0.0477879

Abbreviations: HR hazard ratio; Analysis of a Cox proportional hazards model was performed after adjusting for sex and age upon diagnosis
